# Enantiomeric *Cephalotaxus* alkaloids from seeds of *Cephalotaxus oliveri*

**DOI:** 10.1007/s13659-022-00344-1

**Published:** 2022-07-01

**Authors:** Guang-Xing Yu, Jing Wu, Bao-Bao Shi, Mei-Fen Bao, Xiang-Hai Cai

**Affiliations:** 1 State Key Laboratory of Phytochemistry and Plant Resources in West China, Kunming Institute of Botany, Chinese Academy of Sciences, Kunming, 650201 People’s Republic of China; 2 University of Chinese Academy of Sciences, Beijing, 100039 People’s Republic of China

**Keywords:** *Cephalotaxus oliveri*, Cephalotaxaceae, Alkaloid, Enantiomer

## Abstract

Five undescribed alkaloids were isolated from the seeds of *Cephalotaxus oliveri* along with 27 known ones. The new structures were elucidated based on spectroscopic data including 1D and 2D NMR, MS and calculated ECD spectra. Among them, (+)-acetylcephalofortine C was an enantiomeric *Cephalotaxine* alkaloids. The performed bioassay revealed that those alkaloids were not cytotoxic against cancer cells and had no neuroprotective properties in the HEI-OC-1 cells model.

**Supplementary Information:**

The online version contains supplementary material available at 10.1007/s13659-022-00344-1.

## Introduction

**Fig. 1:**
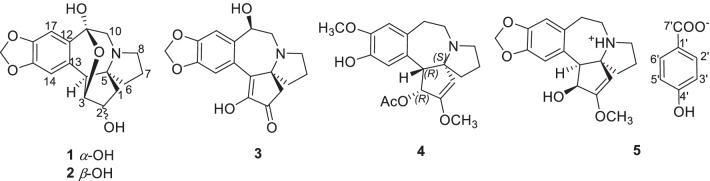
Structures of alkaloids from seeds of *C. oliveri*

The cephalotaxine-type alkaloid was firstly found to possess inhibitory activities against several human tumor cell lines, especially the leukemia cell line [1, 2], consistent with Chinese folk usage. After immense effort of clinical researches, China first approved homoharringtonine as a drug in acute non-lymphocytic leukemia, myelodysplastic syndrome, chronic myelocytic leukemia and polycythemia vera in 1970’s [3, 4]. Recently, homoharringtonine was approved in 2012 by the Food and Drug Administration for the treatment of chronic myeloid leukemia [5]. Plants of the genus *Cephalotaxus* (Cephalotaxaceae) were the only resource of this type of natural products. So the alkaloids from *Cephalotaxus* species have attracted great attention from scientists [6, 7]. Till now, stems and leaves of *Cephalotaxus* have been carried out. Seeds of this genus were also important resources, for example, homoharringtonine was first found from seeds of *C.* *harringtonia* [1]. However, studies of alkaloids in seeds are virgin lands compared to that of stems and leaves. Over the past 60 years, more than 100 alkaloids in *Cephalotaxus* have been reported, which were mainly classified into homoerythrina and the cephalotaxine-types according to their skeleton features [8, 9]. In order to exploit the structure diversity and enrich the library of *Cephalotaxus* alkaloids, phytochemical investigations have been made on the seeds of *C. oliveri*. As a result, five new alkaloids, together with 27 known ones (Fig. 1) were isolated from the seeds of *C. oliveri*. Here we reported the isolation, structure elucidation of those alkaloids.

## Results and discussion

**Fig. 2:**
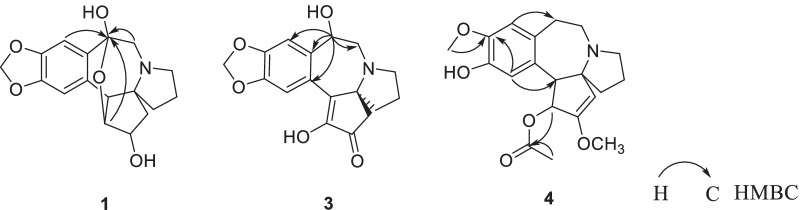
Key HMBC correlations of alkaloids **1**, **3** and **4**

Newly isolates (**1**–**5**) probably belong to alkaloids as they exhibited a positive reaction with Dragendorff’s reagent. Alkaloid **1** was obtained as a white powder. Its molecular formula was determined as C_17_H_19_NO_5_ from HRESIMS *m/z* = 318.1338 [M+H]^+^ (Additional file 1: S5). The ^1^H and ^13^C NMR spectroscopic data (Table 1, Additional file 1: S1~S2) showed the occurrence of two aromatic protons (*δ*_H_ 6.71, 6.92, each s) at para-position, a methylenedioxy moiety (*δ*_H_ 5.88, 5.87), two *O*-bearing CH groups (*δ*_H_ 4.15, *δ*_C_ 76.2; *δ*_H_ 3.72, *δ*_C_ 83.0) and a ketal carbon (*δ*_C_ 98.7). The ^1^H and ^13^C NMR patterns of **1** were very similar to those of the known alkaloid cephalotine A (**17**) [10] with the exception for somewhat differences in chemical shifts values between both alkaloids. The same molecular formulas of both alkaloids assigned their isomeric relationship. H-3 (*δ*_H_ 3.72), H-10 (*δ*_H_ 2.70, 2.51), and H-17 (*δ*_H_ 6.92) showed the HMBC correlations to the ketal carbon (*δ*_C_ 98.7), allowing the ketal carbon at C-11 other than C-3 in **17** (Fig. 2, Additional file 1: S4). Configuration of H-2 was established as *β*-orientation on the basis of the coupling constant (*d*, *J* = 6.6 Hz) of H-2, which was same as that of **17**. Consequently, the structure of **1** was confirmed as shown in Fig. 1, and named isocephalotine A.

**Table 1:** ^1^H (600 MHz) and ^13^C (150 MHz) NMR spectroscopic assignments of **1**–**5** (in methonal-*d*_4_) (*δ* in ppm and *J* in Hz)

Position	*δ*_H_(**1**)	*δ*_C_(**1**)	*δ*_H_(**2**)^a^	*δ*_C_(**2**)^b^	*δ*_H_(**3**)	*δ*_C_(**3**)	*δ*_H_(**4**)	*δ*_H_(3)	*δ*_H_(**5**)	*δ*_C_(**5**)
1	2.30, dd, 15.3, 6.6 1.34, d, 15.3	36.8, CH_2_	1.70, dd, 14.4, 9.2 1.82, dd, 14.4, 9.2	34.5, CH_2_	2.48, d, 18.2 2.36, d, 18.2	50.0, CH_2_	5.15, s	100.6, CH	5.00, s	93.7, CH
2	4.15, d, 6.6	76.2, CH	4.09, td, 9.2, 3.1	74.6, CH		201.3, C		160.1, C		168.2, C
3	3.72, d, 3.7	83.0, CH	3.75, t, 3.1	79.1, CH		151.4, C	5.70, d, 9.2	76.5, CH	4.70, d, 9.2	73.9, CH
4	3.35, d, 3.7	50.9, CH	3.08, d, 3.1	50.5, CH		143.0, C	3.78, d, 9.2	57.0, CH	3.77, d, 9.2	55.8, CH
5		72.3, C		71.5, C		70.4, C		74.4, C		77.6, C
6	1.73, m	38.6, CH_2_	1.68, m 1.61, overlap	38.5, CH_2_	1.65, overlap	38.6, CH_2_	1.96, m 1.89, m	43.7, CH_2_	2.03, overlap	40.9, CH_2_
7	1.62, m	21.4, CH_2_	1.61, overlap	21.3, CH_2_	1.73, m 1.65, overlap	24.0, CH_2_	1.78, m 1.68, m	20.9, CH_2_	1.98, overlap 1.83, m	19.9, CH_2_
8	2.55, m 2.34, overlap	50.2, CH_2_	2.58, m 2.25, m	50.8, CH_2_	2.95, t, 7.8 2.76, m	53.0, CH_2_	2.91, m 2.63, overlap	54.8, CH_2_	3.33, td, 9.5, 7.0 3.09, td, 10.5, 7.0	53.7, CH_2_
10	2.70, d, 20.4 2.51, d, 20.4	60.0, CH_2_	3.23, overlap 2.68, m	60.1, CH_2_	3.15, dd, 15.0, 5.6 3.04, dd, 15.0, 10.5	52.2, CH_2_	2.84, td, 11.8, 7.4 2.63, overlap	49.8, CH_2_	3.17, td, 14.0, 7.0 2.98, td, 11.0, 7.0	48.9, CH_2_
11		98.7, C		99.0, C	4.90, dd, 10.5, 5.6	70.6, CH	3.17, m 2.30, dd, 14.5, 7.4	31.5, CH_2_	3.48, td, 14.0, 8.5 2.40, td, 11.0, 8.5	29.7, CH_2_
12		134.8, C		134.6, C		136.1, C		133.8, C		132.1, C
13		128.5, C		128.9, C		125.1, C		127.2, C		128.2, C
14	6.71, s	109.0, CH	6.93, s	108.7, CH	6.83, s	110.0, CH	6.58, s	119.1, CH	6.70, s	114.3, CH
15		148.9, C		148.7, C		149.6, C		147.0, C		149.1, C
16		148.3, C		148.3, C		148.6, C		147.1, C		148.2, C
17	6.92, s	104.8, CH	6.65, s	104.8, CH	6.95, s	111.5, CH	6.54, s	118.0, CH	6.66, s	111.7, CH
OCH_2_O/16-OCH_3_	5.88, s 5.87, s	102.3, CH_2_	5.87, d, 1.2 5.86, d, 1.2	102.2, CH_2_	5.92, s 5.90, s	103.0, CH_2_			5.83, d, 1.5 5.81, d, 1.5	102.5, CH_2_
2-OCH_3_							3.66, s	58.0, CH_3_	3.71, s	58.3, OCH_3_
CH_3_CO							1.41, s	20.4, CH_3_		
CH_3_CO								171.9, C		

^a^Recorded at 500 MHz

^b^125 MHz

Alkaloid **2** was obtained as a white powder. Its molecular formula C_17_H_19_NO_5_ was same as **1** from its HRESIMS *m/z* 318.1342 [M+H]^+^ (Additional file 1: S11). The 1H and ^13^C NMR data of **2** and **1** (Table 1, Additional file 1: S7~S8) were almost same with exception for ^1^H and ^13^C NMR spectroscopic data of C-1/2/3 between both compounds, suggesting they shared the same planar structure. Obvious differences between both alkaloids were the highfield signals of C-1 (*δ*_C_ 34.5, *Δδ*_C_ − 2.3), C-2 (*δ*_C_ 74.6, *Δδ*_C_ − 1.6) and C-3 (*δ*_C_ 79.1_,_ *Δδ*_C_ − 3.9) in **2**, indicating *α*-configuration of H-2 in **2** as in cephalotine B [10], which was confirmed by the ROESY correlation from H-2 (*δ*_H_ 4.09) to H-4 (*δ*_H_ 3.08) (Additional file 1: S10). Thus, **2** was established as shown in Fig. 1, and named isocephalotine B.

**Fig. 3:**
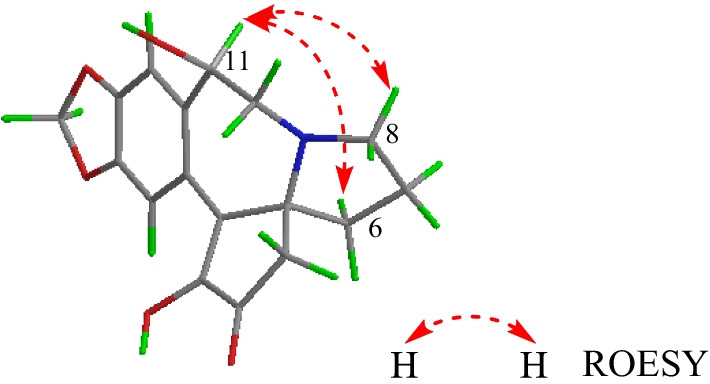
Key ROESY correlations of **3**

Alkaloid **3** was obtained as a white powder. Its molecular formula was determined as C_17_H_17_NO_5_ from HRESIMS *m/z* = 316.1177 [M+H]^+^ (Additional file 1: S18). Alkaloid **3** displayed similar ^1^H and ^13^C NMR data (Table 1, Additional file 1: S13-S14) to the known alkaloid demethylcephalotaxinone (**12**), except that a methylene in **12** was substituted by a methine (*δ*_C_ 70.6, *δ*_H_ 4.90) in **3**. In addition, the HMBC correlations from the methine (*δ*_H_ 4.90) to C-10 (*δ*_C_ 52.2), C-12 (*δ*_C_ 136.1), C-13 (*δ*_C_ 125.1), and C-17 (*δ*_C_ 111.5) located the methine at C-11 (Fig. 2, Additional file 1: S16). H-11 was assigned as *α*-orientated based on the ROESY correlation of H-11 with H-6 (*δ*_H_ 1.65) and H-8 (*δ*_H_ 2.76) (Fig. 3, Additional file 1: S17). Thus, the structure of **3** was established as shown in Fig. 1, and named as 11*β*-hydroxydemethylcephalotaxinone.

**Fig. 4:**
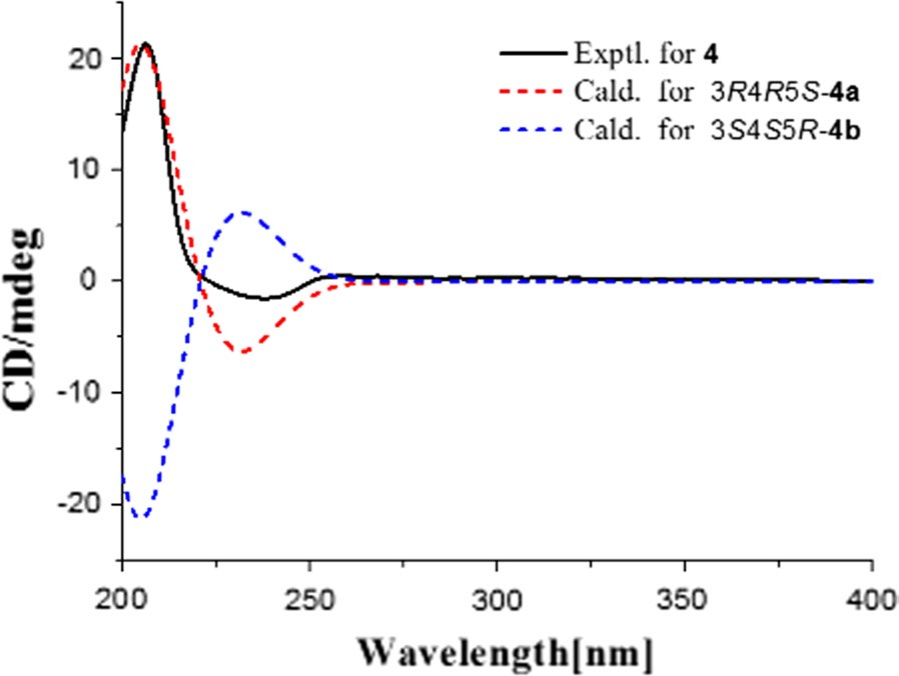
Experimental and calculated ECD spectra of **4**

Alkaloid **4** was obtained as a white powder. Its molecular formula was determined as C_20_H_25_NO_5_ from HRESIMS *m/z* = 360.1807 [M+H]^+^ (Additional file 1: S23). The ^1^H NMR data (Table 1, Additional file 1: S20) were very similar to those of the known alkaloid cephalofortine C [11], with exception for the downfield H-3 signal (*δ*_H_ 5.70, *Δδ*_C_ + 0.97) and a methyl signal (*δ*_H_ 1.41). Additionally, compared to cephalofortine C, **4** had an acetyl group in its ^13^C NMR (*δ*_C_ 20.4, 171.9). The key HMBC correlations from both H-3 (*δ*_H_ 5.70) and CH_3_ (*δ*_H_ 1.41) to carbonyl carbon (*δ*_C_ 171.9) suggested the existence of the acetyl group (Additional file 1: S22). Thus the gross structure of alkaloid **4** was elucidated as shown in Fig. 2. The large coupling constant of H-3/4 (*δ*_H_ 5.70, d, *J* = 9.2 Hz) assigned the configurations of H-3 and H-4 as those of cephalotaxine [12]. However, like to (+)-acetylcephalotaxine ([*α*]_D_ + 102) rather than (−)-acetylcephalotaxine ($[\alpha ]_{{\text{D}}}^{25}$ − 97) [13], **4** might be also assigned to (+)-form based on its specific rotation value ($[\alpha ]_{{\text{D}}}^{23.0}$ + 107.2). Finally, the absolute configuration of **4** was determined by the time-dependent density functional theory ECD calculation. The calculated ECD spectrum of (3*R*,4*R*,5*S*)-**4** was consistent with the experimentally observed ECD spectrum (Fig. 4) (Additional file 1: S32). As a result, the absolute configuration of compound **4** was defined as 3*R*,4*R*,5*S*. Thus, the structure of alkaloid **4** was elucidated as shown in Fig. 1, and named as (+)-acetylcephalofortine C (**4**).

Alkaloid **5** was obtained as a colorless crystal. Analysis of the ^1^H and ^13^C NMR spectra (Table 1, Additional file 1: S25~S26) indicated the presence of following signals, a 1,2,4,5-tetra substituted benzenoid ring with a methylenedioxy substitution (*δ*_H_ 6.70, 6.66, 5.83, 5.81), five methylenes (*δ*_C_ 40.9, 19.9, 53.7, 48.9, 29.7), three methines (*δ*_C_ 93.7, 73.9, 55.8), two quaternary carbons (*δ*_C_ 77.6, 168.2) and one methoxy (*δ*_C_ 58.3), which were agreed well with signals of cephalotaxine. Its molecular formula C_18_H_21_NO_4_ from HRESIMS *m/z* 316.1549 [M+H]^+^ (Additional file 1: S29) was also same with that of cephalotaxine. The rest of C-1′ (*δ*_C_ 128.9), C-2′ (*δ*_C_ 132.4, *δ*_H_ 7.71), C-3′ (*δ*_C_ 115.3, *δ*_H_ 6.65), C-4′ (*δ*_C_ 161.2) and a carbonyl group (*δ*_C_ 174.6) signals revealed a *p*-hydroxybenzoic acid structure, which was supported by the HMBC correlations from H-2′ to C-3′, C-4′, the carbonyl group and from H-3′ to C-4′ (Fig. 2, Additional file 1: S28). Its molecular formula C_7_H_6_O_3_ was revealed by the HRESIMS *m/z* = 137.0238 [M−H]^−^ (Additional file 1: S30). Carefully analyzing the ^13^C NMR spectra data of **5**, revealed the upfield signals of C-1 (*δ*_C_ 93.7), C-4 (*δ*_C_ 55.8), C-6 (*δ*_C_ 40.9) and the downfield signal of C-5 (*δ*_C_ 77.6). These changes were consistent with the ^13^C NMR signals of cephalotaxine ester salts [14], which means compound **5** was a *p*-hydroxybenzoate salt of cephalotaxine. The specific rotation ($[\alpha ]_{{\text{D}}}^{23.5}$ − 216.4) of **5** was close to that of (−)-cephalotaxine ($[\alpha ]_{{\text{D}}}^{25}$ − 204). Thus, the structure of **5** was established as (−)-cephalotaxine *p*-hydroxybenzoate.

The known alkaloids were identified as 11-hydroxycephalotaxine (**6**) [15], cephalotaxine (**7**) [16], acetylcephalotaxine (**8**) [17], drupacine (**9**) [17], 3-deoxy-3,11-epoxy- cephalotaxine (**10**) [18], drupacinamide (**11**) [19], demethylcephalotaxinone (**12**) [20], cephalotaxinone (**13**) [21], 11β-hydroxycephalotaxine *N*-oxide (**14**) [22], cephalotine D (**15**) [10], cephalancetine C (**16**) [23], cephalotine A (**17**) [10], cephalotine B (**18**) [10], cephalocyclidin A (**19**) [24], cephalofortunone (**20**) [25], deoxyharringtonine (**21**) [2], isoharringtonine (**22**) [1, 26], 3'-*R*-isoharringtonine (**23**) [26], harringtonine (**24**) [1], deoxyharringtonic acid (**25**) [13], 5'-des-*O*-methylisoharringtonine (**26**) [13], 5'-des-*O*-methylharringtonine (**27**) [27], nordeoxyharringtonine (**28**) [28], epischellhammericine B (**29**) [29], 3-epicomosine (**30**) [30], 3-epischellhammericine (**31**) [29], taxodinea (**32**) [31] by comparison with literatures.

In biological activity research, none of these new compounds showed cytotoxic activity against HeLa, SGC-7901 gastric cancer, and A-549 lung cancer cell lines (IC_50_ > 20 µM). Additionally, unlike homologous *Erythrina* alkaloids [32], all of them also had no neuroprotective effects on HEI-OC-1 cells model.

Alkaloids **1**–**5** were undescribed compounds. Interestingly, the **4** was assigned as rare (+)-form enantiomer. This was the first to report alkaloids from the seeds of *C. oliveri*.

## Experimental section

### General experimental procedures

Optical rotations were measured with a RUDOLPH APVI-6 automatic polarimeter. UV spectra were recorded on a Shimadzu 2401A spectrophotometer. 1D and 2D NMR spectra were acquired Bruker AVANCE III-600 MHz spectrometers with SiMe4 as an internal standard. MS data were obtained using a Shimadzu UPLC-IT-TOF (Shimadzu Corp., Kyoto, Japan). Column chromatography (CC) was performed on either silica gel (200–300 mesh, Qing-dao Haiyang Chemical Co., Ltd., Qingdao, China) or RP-18 silica gel (20–45 um, Fuji Silysia Chemical Ltd., Japan). Fractions were monitored by TLC on silica gel plates (GF254, Qingdao Haiyang Chemical Co., Ltd., Qingdao, China), and spots were visualized with Dragendorff’s reagent spray. MPLC was performed using a Buchi pump system coupled with RP-18 silica gel-packed glass columns (19 × 480, 40 × 480, 45 × 480, and 55 × 480 mm, respectively). HPLC was performed using Waters 1525EF pumps coupled with analytical semi-preparative or preparative Xbridge C_18_ columns (4.6 × 150 and 19 × 250 mm, respectively). The HPLC system employed a Waters 2998 photodiode array detector and a Waters fraction collector III.

### Plant materials

Seeds of *Cephalotaxus oliveri* Mast. (Cephalotaxaceae) were collected in October 2013 in Yunnan Province, P. R. China and identified by Xiang-hai Cai. The voucher specimen (cai20131002) was preserved in the State Key Laboratory of Phytochemistry and Plant Resources in West China, Kunming Institute of Botany, Chinese Academy of Sciences.

### Extraction and isolation

The air-dried and powdered seeds of *C. oliveri* (14 kg) were extracted with MeOH at room temperature, and the solvent was evaporated in vacuo. The crude extract was suspended in HCl (1%) and partitioned with Petroleum ether and EtOAc. Then the acidic layer was adjusted to pH 7–8 with 5% ammonia solution and subsequently extracted with EtOAc to obtain crude alkaloid extract (45 g). The crude alkaloids was subjected to CC over silica gel (400 g) and eluted with a CHCl_3_–MeOH gradient (1:0 to 0:1, v/v) to give five fractions (I–V) based on TLC analysis.

Fraction I (10 g) was subjected to C_18_ MPLC with MeOH–H_2_O (1:9 to 100:0, v/v) as the eluent to obtain eight fractions (I-1 ~ 8). Subfraction I-1 ~ 2 were identified as **9** (4.5 g) by ^1^H-NMR. Subfraction I-3 was purified by a Sephadex LH-20 column and eluted with MeOH to obtain two fractions (I-3-1 ~ 2). Subfraction I-3-1 was then separated on a preparative C_18_ column with a gradient CH_3_CN–H_2_O (1:4 to 7:13, v/v) to afford **25** (3.6 mg), **26** (11.5 mg). Subfraction 1-3-2 was separated on a preparative C_18_ column with a gradient CH_3_CN–H_2_O (3:7 to 9:11, v/v) to afford **20** (1 mg); Subfraction I-4 was purified by a Sephadex LH-20 column and eluted with MeOH to obtain **22** (23 mg). Subfraction I-5 was purified by a Sephadex LH-20 column (eluted with MeOH) and then separated on a preparative C_18_ column with a gradient CH_3_CN–H_2_O (3:7 to 9:11, v/v) to afford **8** (41.3 mg). Subfraction 1–6 was identified as **22** (1 g) by ^1^H-NMR. Subfraction I-7 was purified by a Sephadex LH-20 column (eluted with MeOH) and then separated on a preparative C_18_ column with a gradient CH_3_CN–H_2_O (9:11 to 3:2, v/v) to afford **28** (21 mg) and **31** (66.3 mg). Subfraction I-8 was identified as **21** (2.5 g) by ^1^H-NMR.

Fraction II (2 g) was subjected to CC over silica gel (20 g) and eluted with a CHCl_3_–MeOH gradient (1:0 to 0:1, v/v) to give two fractions (II-1 ~ 2). Subfraction II-1 was purified by a Sephadex LH-20 column and eluted with MeOH to obtain two fractions (II-1-1 ~ 2). Subfraction II-1-1 was identified as **22** (0.6 g) by ^1^H-NMR. Subfraction II-1-2 was subjected to C_18_ MPLC with MeOH–H_2_O (1:9 to 100:0, v/v) as the eluent and then separated on a preparative C_18_ column with a gradient CH_3_CN–H_2_O (1:4 to 7:13, v/v) to afford **11** (1.4 mg), **16** (2 mg), **29** (24.6 mg), **30** (15.7 mg). Subfraction II-2 was purified by a Sephadex LH-20 column and eluted with MeOH to obtain **7** (93.8 mg), the rest part was then separated on a preparative C_18_ column with a gradient CH_3_CN–H_2_O (1:4 to 7:13, v/v) to afford **26** (11.5 mg).

Fraction III (1 g) was subjected to C_18_ MPLC with MeOH–H_2_O (1:9 to 100:0, v/v) as the eluent to obtain three fractions (III-1 ~ 3). Subfraction III-1 was identified as** 7** (90.0 mg) by ^1^H-NMR. Subfraction III-2 was identified as **22** (162 mg) by ^1^H-NMR. Subfraction III-3 was purified by a Sephadex LH-20 column and eluted with MeOH to obtain two fractions (III-3-1 ~ 2). Subfraction III-3-1 was then separated on a preparative C_18_ column with a gradient CH_3_CN–H_2_O (1:4 to 7:13, v/v) to afford **26** (8 mg). Subfraction III-3-2 was then separated on a preparative C_18_ column with a gradient CH_3_CN–H_2_O (9:11 to 3:2, v/v) to afford **23** (23.1 mg).

Fraction IV (0.7 g) was subjected to C_18_ MPLC with MeOH–H_2_O (1:9 to 100:0, v/v) as the eluent to obtain three fractions (IV-1 ~ 3). Subfraction IV-1 was purified by a Sephadex LH-20 column and eluted with MeOH to obtain **7** (550 mg). Subfraction IV-2 was purified by a Sephadex LH-20 column (eluted with MeOH) and then separated on a preparative C_18_ column with a gradient CH_3_CN–H_2_O (2:3 to 11:9, v/v) to afford **24** (51.5 mg). Subfraction IV-3 was purified by a Sephadex LH-20 column to gain **7** (40 mg).

Fraction V (25 g) was subjected to C_18_ MPLC with MeOH–H_2_O (1:19 to 100:0, v/v) as the eluent to obtain four fractions (V-1 ~ 4). Subfraction V-1 was purified by C_18_ MPLC with MeOH–H_2_O (1:19 to 100:0, v/v) as the eluent to obtain three fractions (V-1-1 ~ 3). Subfraction V-1-1 separated on a preparative C_18_ column with a gradient CH_3_CN–H_2_O (1:9 to 1:3, v/v) to afford **19** (4.7 mg), **18** (1 mg) and **17** (2.2 mg); Subfraction V-1-2 was then purified by a Sephadex LH-20 column and eluted with MeOH to obtain **5** (1.5 g). Subfraction V-1-3 was then purified by a Sephadex LH-20 column and eluted with MeOH to obtain **7** (7.3 g). Subfraction V-2 was purified by C_18_ MPLC with MeOH–H_2_O (1:19 to 100:0, v/v) as the eluent and then separated on a preparative C_18_ column with a gradient CH_3_CN–H_2_O (3:7 to 9:11, v/v) to afford **10** (1.1 mg). Subfraction V-3 was subjected to C_18_ MPLC with MeOH–H_2_O (1:19 to 100:0, v/v) as the eluent to obtain five fractions (V-3-1 ~ 5). Subfraction V-3-1 was purified by a Sephadex LH-20 column and eluted with MeOH to obtain two fractions (V-3-1-1 ~ 2). Subfraction V-3-1-1 was identified as **10** (560.9 mg). Subfraction V-3-1-2 was then separated on a preparative C_18_ column with a gradient CH_3_CN–H_2_O (3:17 to 3:7, v/v) to afford **1** (1.9 mg), **2** (5.5 mg) and **13** (0.5 mg). Subfraction V-3-2 was purified by a Sephadex LH-20 column and eluted with MeOH to obtain five fractions (V-3-2-1 ~ 5). Subfraction V-3-2-1 was then separated on a preparative C_18_ column with a gradient CH_3_CN–H_2_O (1:3 to 2:3, v/v) to afford **4** (4.6 mg). Subfraction V-3-2-2 was then separated on a preparative C_18_ column with a gradient CH_3_CN–H_2_O (3:7 to 9:11, v/v) to afford **32** (3.9 mg), **15** (10.4 mg). Subfraction V-3-2-3 was identified as **12** (55.1 mg) by ^1^H-NMR. Subfraction V-3-2-4 was separated on a preparative C_18_ column with a gradient CH_3_CN–H_2_O (1:3 to 2:3, v/v) to afford **3** (7.6 mg), **12** (13.0 mg). Subfraction V-3-3 was purified by a Sephadex LH-20 column and eluted with MeOH and separated on a preparative C_18_ column with a gradient CH_3_CN–H_2_O (1:3 to 2:3, v/v) to obtain **14** (2.5 mg) and **32** (5.6 mg). Subfraction V-3-4 was purified by a Sephadex LH-20 column and eluted with MeOH and separated on a preparative C_18_ column with a gradient CH_3_CN–H_2_O (7:13 to 1:1, v/v) to obtain **6** (302 mg). Subfraction V-3-5 was purified by a Sephadex LH-20 column and eluted with MeOH to obtain **26** (68.8 mg). Subfraction V-4 was purified by C_18_ MPLC with MeOH–H_2_O (1:19 to 100:0, v/v) as the eluent to obtain two fractions (V-4 ~ 1–2). Subfraction V-4-1 was separated on a preparative C_18_ column with a gradient CH_3_CN–H_2_O (1:3 to 2:3, v/v) to obtain **27** (6.3 mg). Subfraction V-4-2 was purified by a Sephadex LH-20 column and eluted with MeOH to obtain **24** (98.4 mg).

Isocephalotine A (**1**): White powder; C_17_H_19_NO_5_; $[\alpha ]_{{\text{D}}}^{23.2}$ − 30.67 (c 0.03, CH_3_OH); UV (CH_3_OH) *λ*_max_ (log*ε*): 203.6 (3.77), 228.4 (3.06), 289.2 (2.95) (Additional file 1: S6); ^1^H (600 MHz) and ^13^C (150 MHz) NMR data (methanol-*d*_4_), see Table 1; positive HRESIMS *m/z* 318.1338 [M+H]^+^ (calcd. for C_17_H_20_NO_5_, 318.1342).

Isocephalotine B (**2**): White powder; C_17_H_19_NO_5_; $[\alpha ]_{{\text{D}}}^{23.4}$ − 26.0 (c 0.11, CH_3_OH); UV (CH_3_OH) *λ*_max_ (log*ε*): 203.6 (3.08), 231.2 (3.30), 290.0 (3.08) (Additional file 1: S12); ^1^H (500 MHz) ^13^C (125 MHz) NMR data (methanol-*d*_4_), see Table 1; positive HRESIMS *m/z* 318.1342 [M+H]^+^ (calcd. for C_17_H_20_NO_5_, 318.1342).

11*β*-Hydroxydemethylcephalotaxinone (**3**): White powder; C_17_H_17_NO_5_; $[\alpha ]_{{\text{D}}}^{22.7}$ 250.5 (c 0.04, CH_3_OH); UV (CH_3_OH) *λ*_max_ (log*ε*): 202.8 (3.48), 251.4 (3.03), 298.8 (3.15) (Additional file 1: S19); ^1^H (600 MHz) and ^13^C (150 MHz) NMR data (methanol-*d*_4_), see Table 1; positive HRESIMS *m/z* 316.1177 [M+H]^+^ (calcd. for C_17_H_18_NO_5_, 316.1179).

(+)-Acetylcephalofortine C (**4**): White powder; C_20_H_25_NO_5_; $[\alpha ]_{{\text{D}}}^{23.0}$ 107.2 (c 0.05, CH_3_OH); UV (CH_3_OH) *λ*_max_ (log*ε*): 204.2 (3.94), 226.4 (3.23), 283.8 (2.79) (Additional file 1: S24); ^1^H (600 MHz) and ^13^C (150 MHz) NMR data (methanol-*d*_4_), see Table 1; positive HRESIMS *m/z* 360.1807 [M+H]^+^ (calcd. for C_20_H_26_NO_5_, 360.1811).

Cephalotaxine *P*-hydroxybenzoate (**5**): $[\alpha ]_{{\text{D}}}^{23.5}$ − 216.4 (c 0.05, CH_3_OH); UV (CH_3_OH) *λ*_max_ (log *ε*): 203.6 (4.17), 246.2 (3.45), 286.2 (3.08) (Additional file 1: S31); ^1^H (500 MHz) and ^13^C (125 MHz) NMR data (methanol-*d*_4_) *δ*_C_: 128.9 (s, C-1′), 132.4 (d, C-2′/6′), 115.3 (d, C-3′/5′), 161.2 (d, C-4′), 174.6 (s, C-7′), other data see Table 1; positive HRESIMS *m/z* 316.1549 [M+H]^+^ (calcd. for C_18_H_22_NO_4_, 316.1549); negative HRESIMS *m/z* 137.0238 [M+H]^+^ (calcd. for C_7_H_5_NO_3_, 137.0239).

### Cytotoxicity assay

The cytotoxicity of compounds **1–5** was tested by the MTS assay. The cells were seeded into 96-well tissue culture dishes at 5 × 10^3^ cells/well for SMMC-7721, HT-29 and A-549 and cultured overnight at 37 °C in a 5% CO_2_ incubator for cell adhesion. Cells were then incubated in culture medium with each compound for 48 h. The MTS-reducing activity was evaluated by measuring the absorbance at 490 nm using the CellTiter 96 Aqueous One Solution Cell Proliferation Assay kit (Promega, USA) and an Infinite M200 Pro (Tecan, Austria) microplate reader. IC_50_ values were calculated using Reed–Muench method.

### Protecting hearing loss assay

HEI-OC1 cells were seeded into 96-well tissue culture dishes at 5 × 10^3^ cells/well and cultured overnight in DMEM medium supplemented with 10% FBS and 50 µg/mL ampicillin. The cells were then incubated with different doses of compounds in DMEM without FBS for 12 h before neomycin exposure. After this pre-treatment, the experimental group was treated with 20 mM neomycin together with compounds, the neomycin-only group was treated with 20 mM neomycin and an equivalent volume of DMSO, and the control group was treated with equivalent volume of DMSO without neomycin or compounds. After another 24 h of culture, the cells were thoroughly washed with PBS and cultured in DMEM with ampicillin for an additional 12 h recovery. The cell viability was then measured using the CellTiter 96 AQueous One Solution Cell Proliferation Assay kit (Promega, USA).

## Supplementary Information

**Additional file 1:** Enantiomeric Cephalotaxus Alkaloids from Seeds of *C. oliveri*. General NMR, HRESIMS, UV and ECD spectra of compound 1-5 and computational methods for the ECD of compound 4.
