# Supplementary material for: Enantiomeric Cephalotaxus alkaloids from seeds of Cephalotaxus oliveri
Source: Nat Prod Bioprospect. 2022 Jul 1;12(1):24. doi: 10.1007/s13659-022-00344-1 (PMC9249953; doi:10.1007/s13659-022-00344-1)
Supplement: Supplementary file 1 — Additional file 1: Enantiomeric Cephalotaxus Alkaloids from Seeds of C. oliveri. General NMR, HRESIMS, UV and ECD spectra of compound 1-5 and computational methods for the ECD of compound 4. [file 13659_2022_344_MOESM1_ESM.pdf]

Enantiomeric *Cephalotaxus* Alkaloids from Seeds of *C. oliveri*

Guang-Xing Yu[a,b], Jing-Wu[a], Bao-Bao Shi[a], Mei-Fen Bao[a], and Xiang-Hai Cai[a,]*

[a]State Key Laboratory of Phytochemistry and Plant Resources in West China, Kunming Institute of Botany, Chinese Academy of Sciences, Kunming 650201, People's Republic of China

[b]University of Chinese Academy of Sciences, Beijing 100039, People's Republic of China

*Corresponding Author. xhcai@mail.kib.ac.cn. Tel: +86-871-65223242; Fax: +86-871-65150227

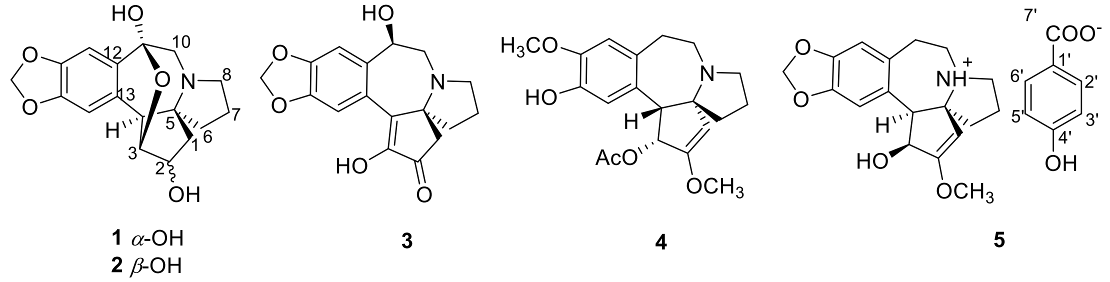

Structures of alkaloids from seeds of *C. oliveri*.

CONTENTS

S 1 ^{1}H NMR spectrum of 1 in Methanol-d_4 ... 3
S 2 ^{13}C and DEPT NMR spectrum of 1 in Methanol-d_4 ... 4
S 3 HSQC spectrum of 1 in Methanol-d_4 ... 5
S 4 HMBC spectrum of 1 in Methanol-d_4 ... 6
S 5 HRMS(ESI) of 1 ... 7
S 6 UV of 1 in MeOH ... 8
S 7 ^{1}H NMR spectrum of 2 in Methanol-d_4 ... 9
S 8 ^{13}C and DEPT spectrum of 2 in Methanol-d_4 ... 10
S 9 HSQC spectrum of 2 in Methanol-d_4 ... 11
S 10 ROESY spectrum of 2 in Methanol-d_4 ... 12
S 11 HRMS(ESI) of 2 ... 13
S 12 UV of 2 in MeOH ... 14
S 13 ^{1}H NMR spectrum of 3 in Methanol-d_4 ... 15
S 14 ^{13}C and DEPT spectrum of 3 in Methanol-d_4 ... 16
S 15 HSQC spectrum of 3 in Methanol-d_4 ... 17
S 16 HMBC spectrum of 3 in Methanol-d_4 ... 18
S 17 ROESY spectrum of 3 in Methanol-d_4 ... 19
S 18 HRMS(ESI) of 3 ... 20
S 19 UV of 3 in MeOH ... 21
S 20 ^{1}H NMR spectrum of 4 in Methanol-d_4 ... 22
S 21 ^{13}C spectrum of 4 in Methanol-d_4 ... 23
S 22 HMBC of 4 in Methanol-d_4 ... 24
S 23 HRMS(ESI) of 4 ... 25
S 24 UV of 4 in MeOH ... 26
S 25 ^{1}H NMR spectrum of 5 in Methanol-d_4 ... 27
S 26 ^{13}C and DEPT spectrum of 5 in Methanol-d_4 ... 28
S 27 HSQC spectrum of 5 in Methanol-d_4 ... 29
S 28 HMBC spectrum of 5 in Methanol-d_4 ... 30
S 29 HRMS(ESI)$^{+}$ of 5 ... 31
S 30 HRMS(ESI)$^{-}$ of 5 ... 32
S 31 UV of 5 in MeOH ... 33
S 32 ECD Computational details of compound 4 ... 34

S 1 ^{1}H NMR spectrum of 1 in Methanol-d_4

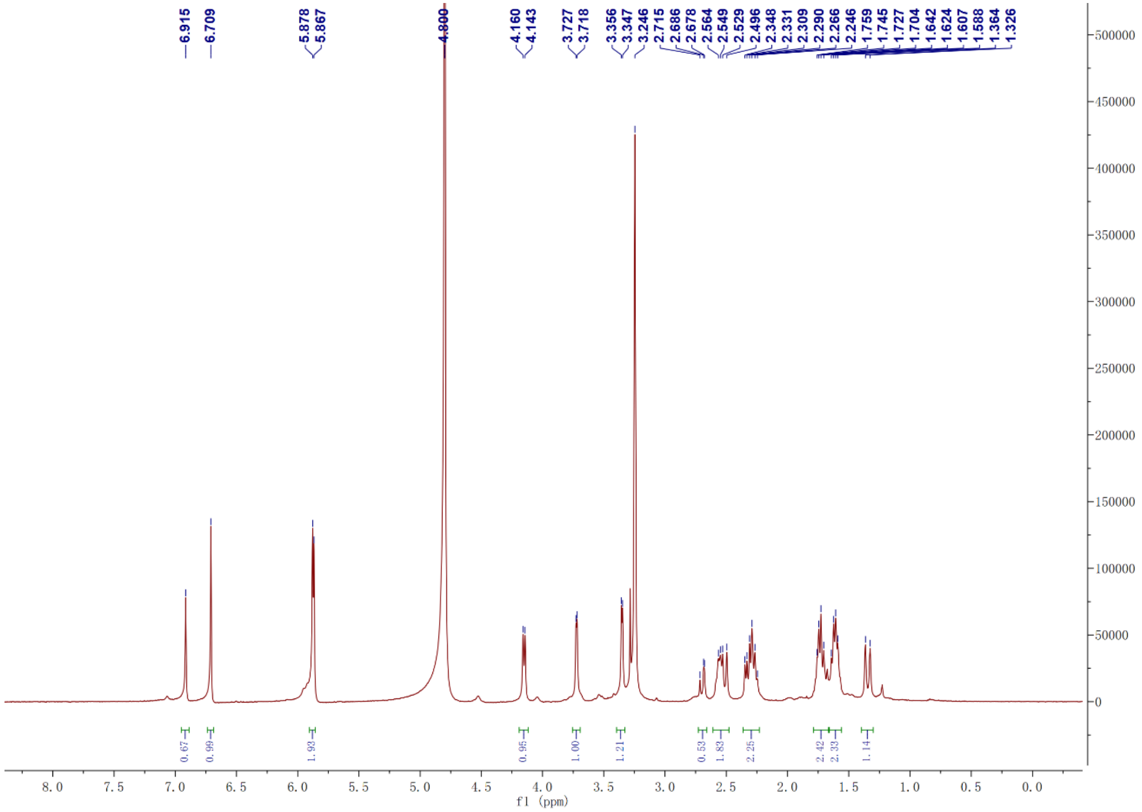

S 2 ^{13}C and DEPT NMR spectrum of 1 in Methanol-d_4

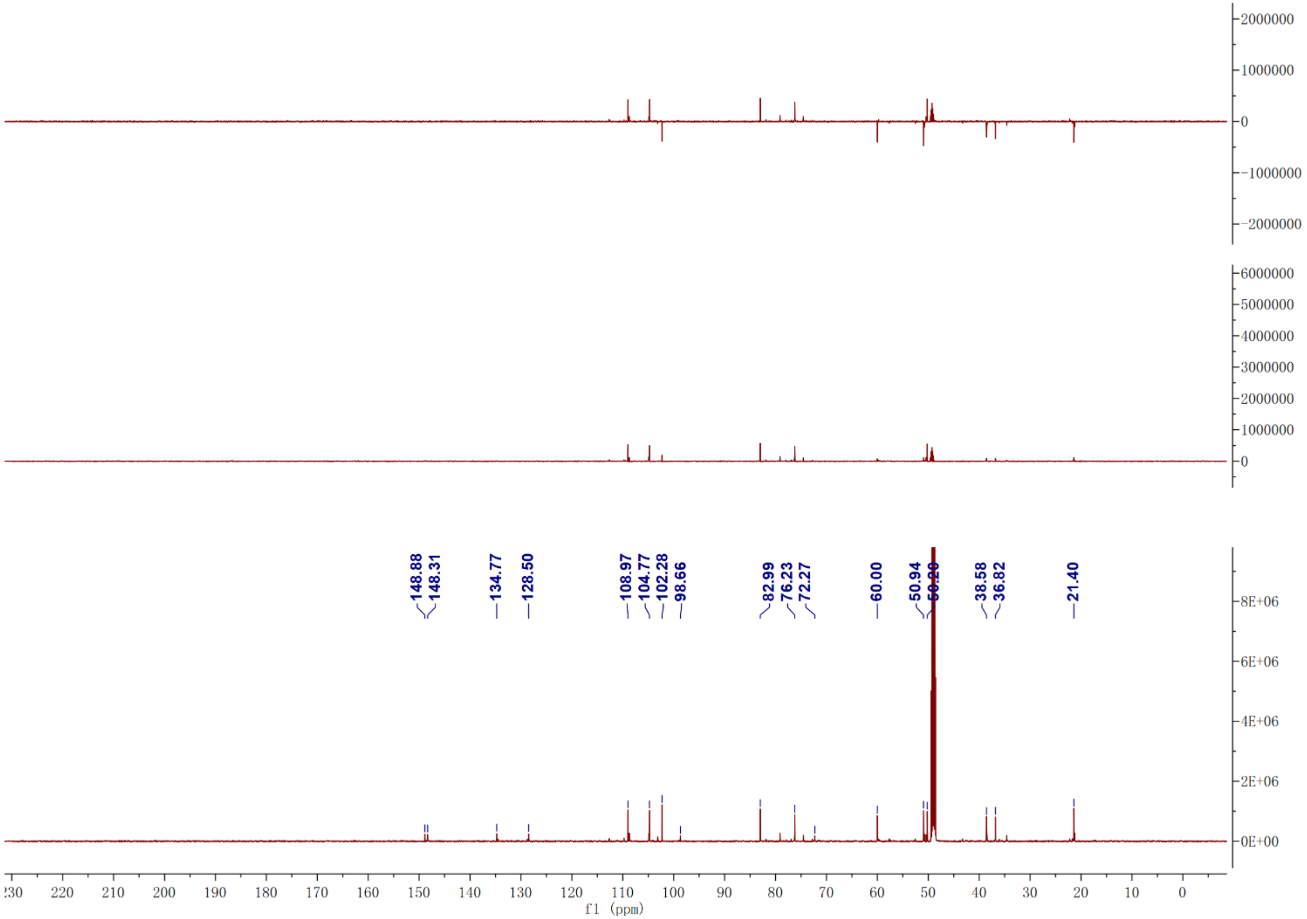

S 3 HSQC spectrum of 1 in Methanol-d_4

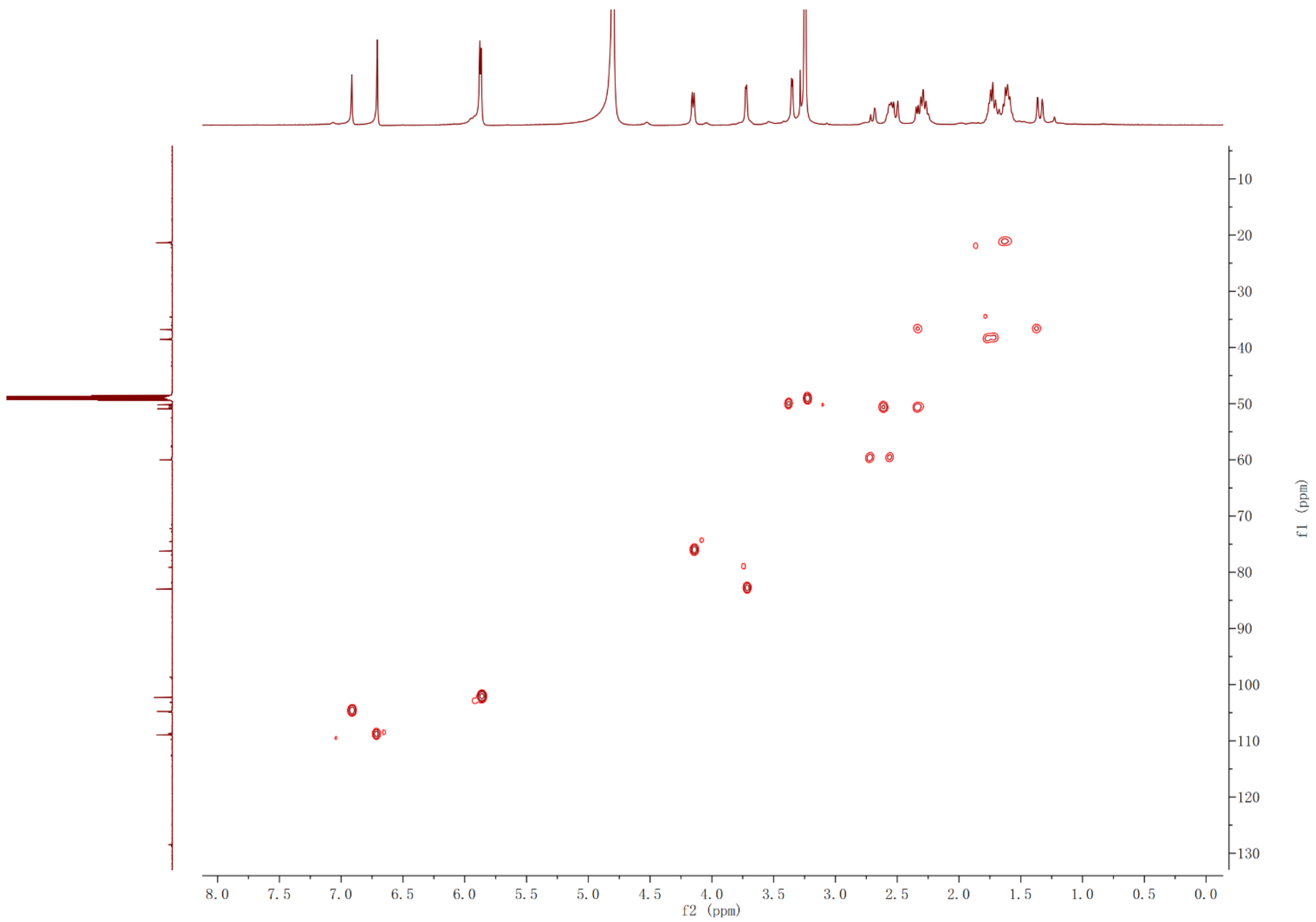

S 4 HMBC spectrum of 1 in Methanol-d_4

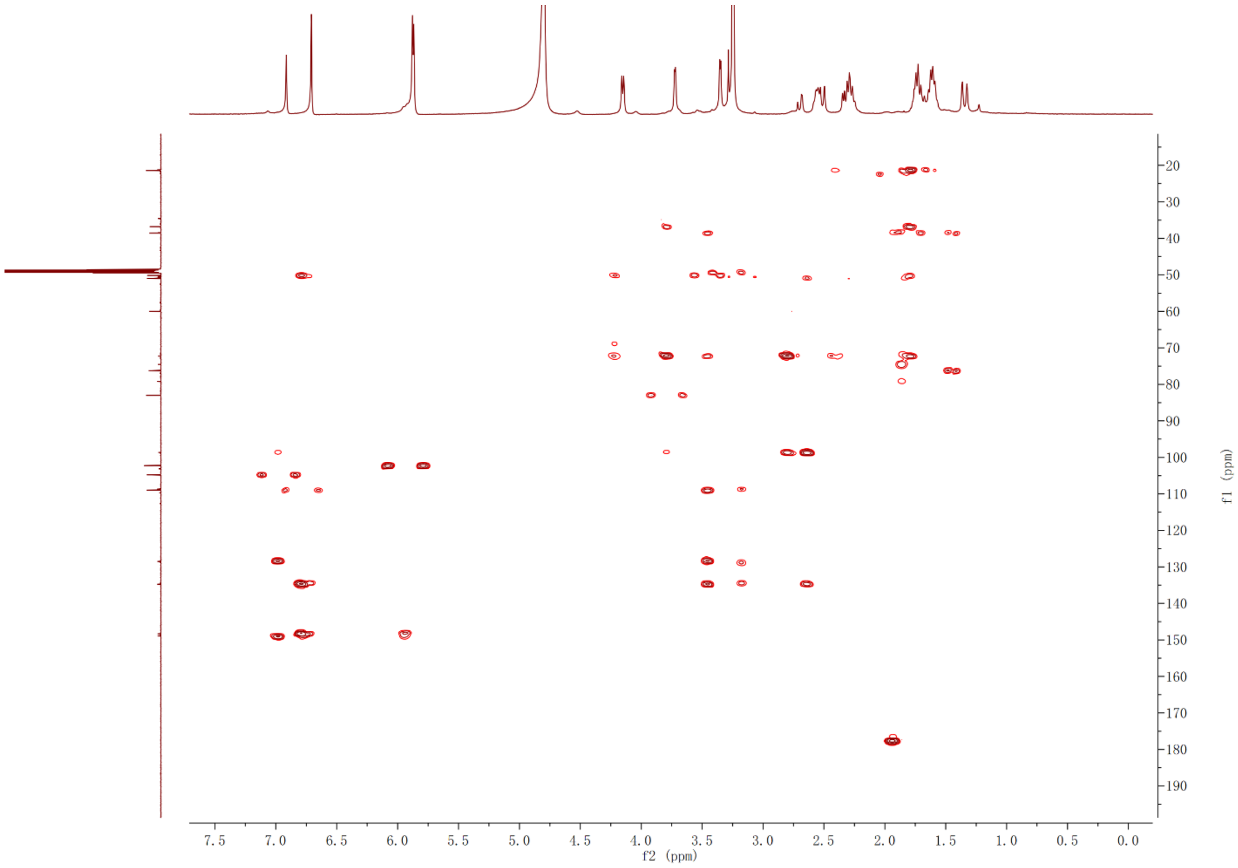

S 5 HRMS(ESI) of 1

Qualitative Analysis Report

**Data Filename**	20211119ESIA6.d	**Sample Name**	wcof42-1
**Sample Type**	Sample	**Position**	
**Instrument Name**	Agilent G6230 TOF MS	**User Name**	KIB
**Acq Method**	ESI.m	**Acquired Time**	11/19/2021 10:25:49 AM
**IRM Calibration Status**	Success	**DA Method**	ESI.m
**Comment**	+		

Sample Group **Info.**

Acquisition SW Version 6200 series TOF/6500 series Q-TOF B.05.01 (B5125.2)

User Spectra

Fragmentor Voltage	Collision Energy	Ionization Mode
200	0	ESI

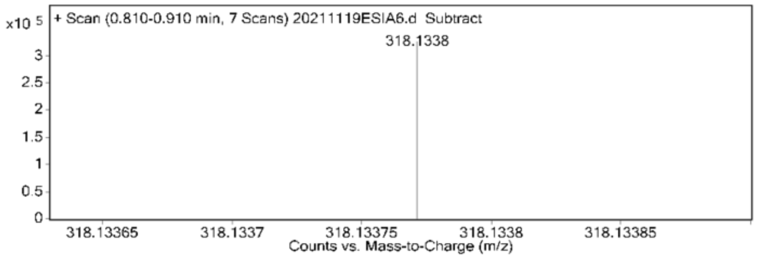

Peak List

m/z	z	Abund	Formula	Ion
177.0538		7503.54		
301.1368	1	9568.22		
316.154	1	53348.08		
317.1572	1	10240.07		
318.1338		325393.72	C17 H20 N O5	M+
319.1398		664021.06		
320.1452	1	333922.63		
321.1483	1	53385.89		
368.4248	1	13741.29		
507.3282		7650.29		

Formula Calculator Element Limits

Element	Min	Max
C	0	200
H	0	400
O	0	10
N	1	1

Formula Calculator Results

Formula	CalculatedMass	Mz	Diff.(mDa)	Diff. (ppm)	DBE
C17 H20 N O5	318.1342	318.1338	0.3	1.1	8.5

--- End Of Report ---

S 6 UV of 1 in MeOH

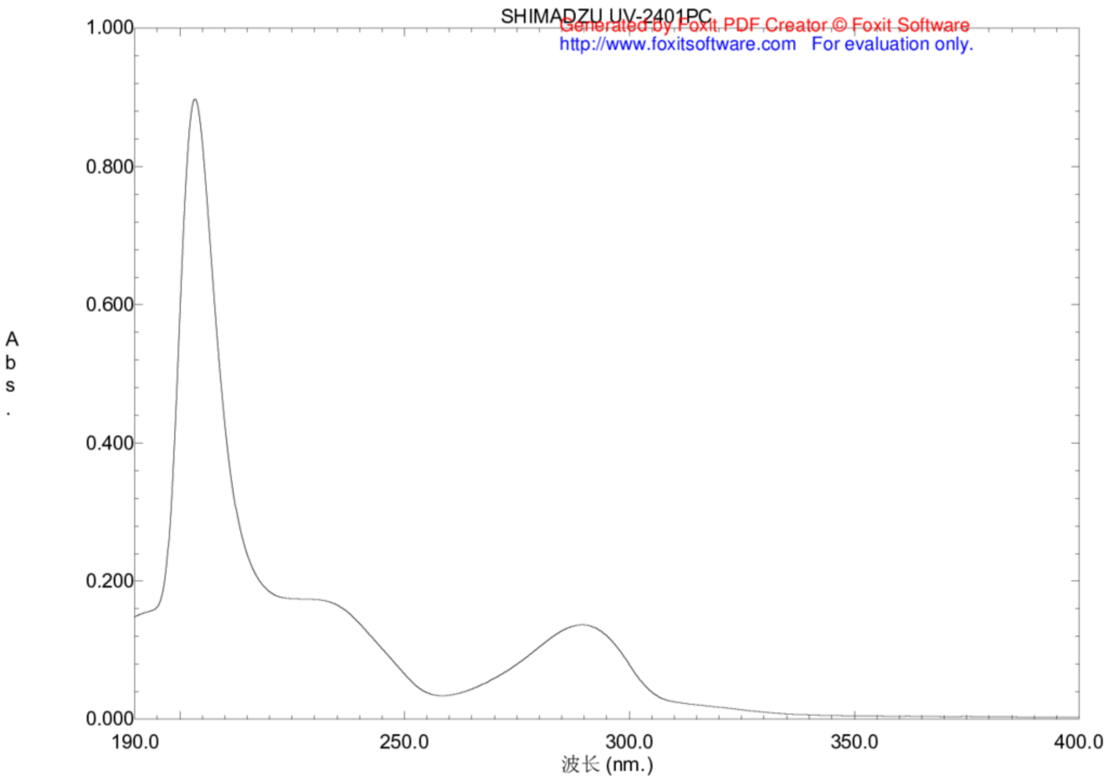

Generated by Foxit PDF Creator © Foxit Software
http://www.foxitsoftware.com For evaluation only.

文件名: WCOF42-1

创建于: 16:09 22-03-16
数据: 原始

测量模式: Abs.
扫描速度: 中速
狭缝: 5.0
采样间隔: 0.2

WCOF42-1

样品浓度：0.0097毫克/毫升
溶剂：甲醇

否.	波长 (nm.)	Abs.
1	203.60	0.8965
2	228.40	0.1734
3	289.20	0.1363

S 7 ^{1}H NMR spectrum of 2 in Methanol-d_4

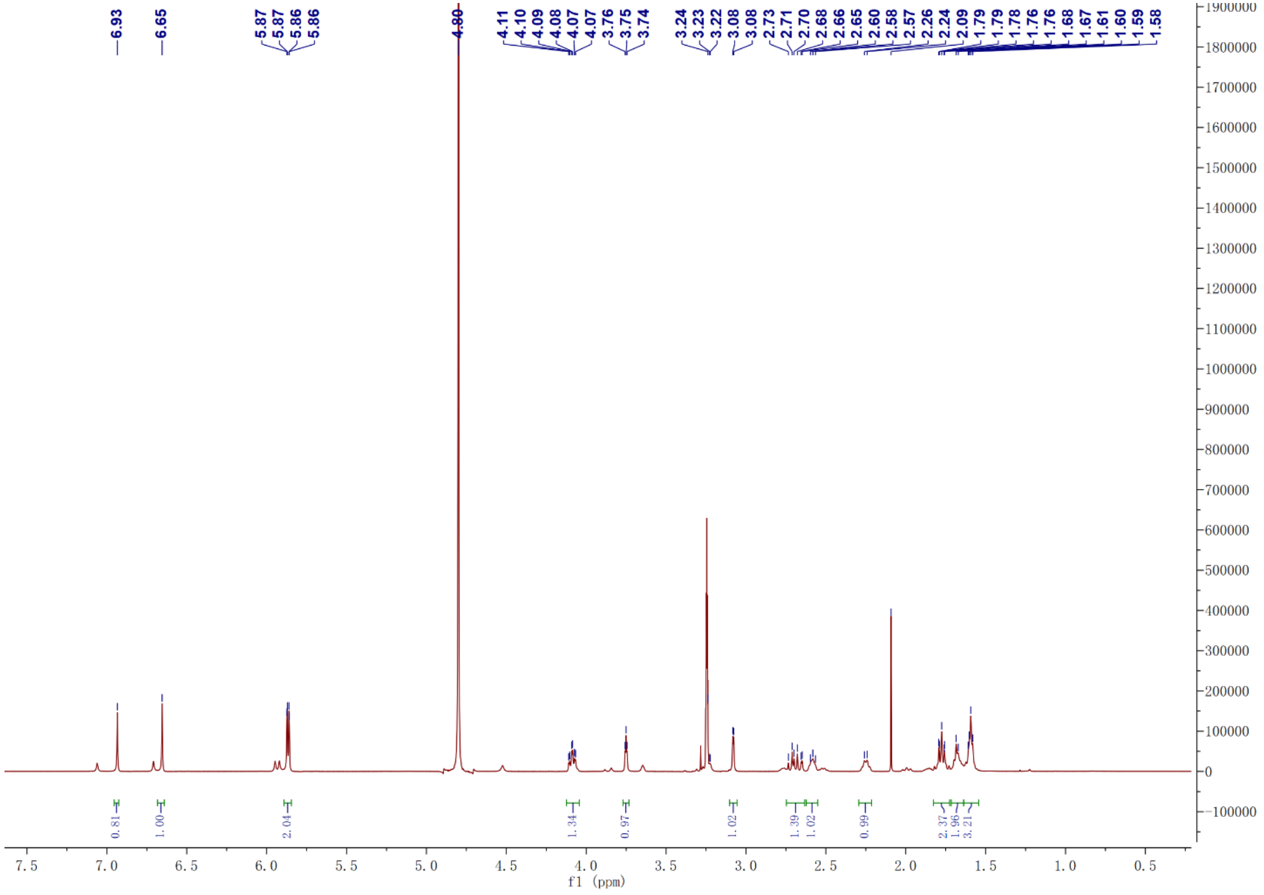

S 8 ^{13}C and DEPT spectrum of 2 in Methanol-d_4

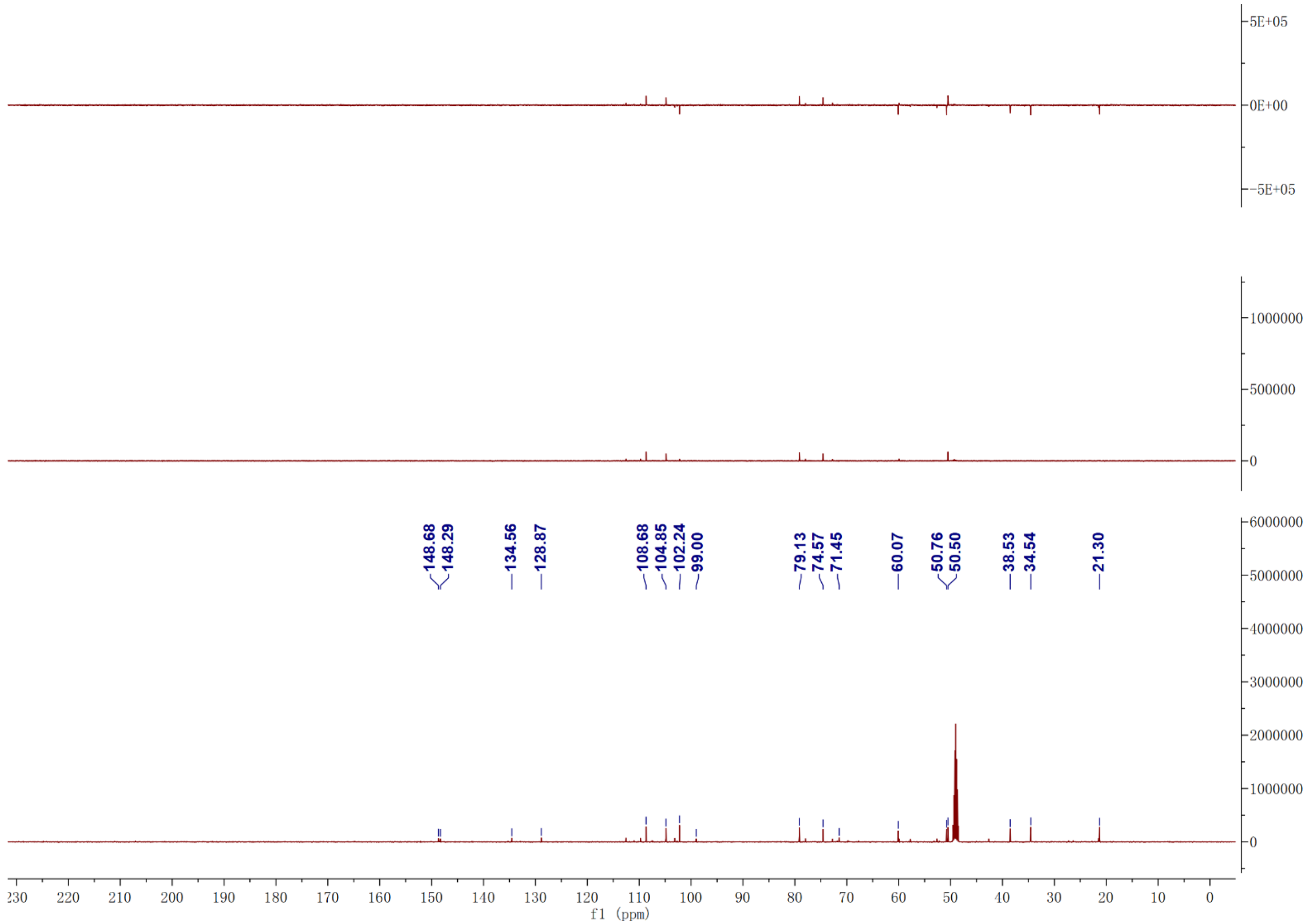

S 9 HSQC spectrum of 2 in Methanol-d_4

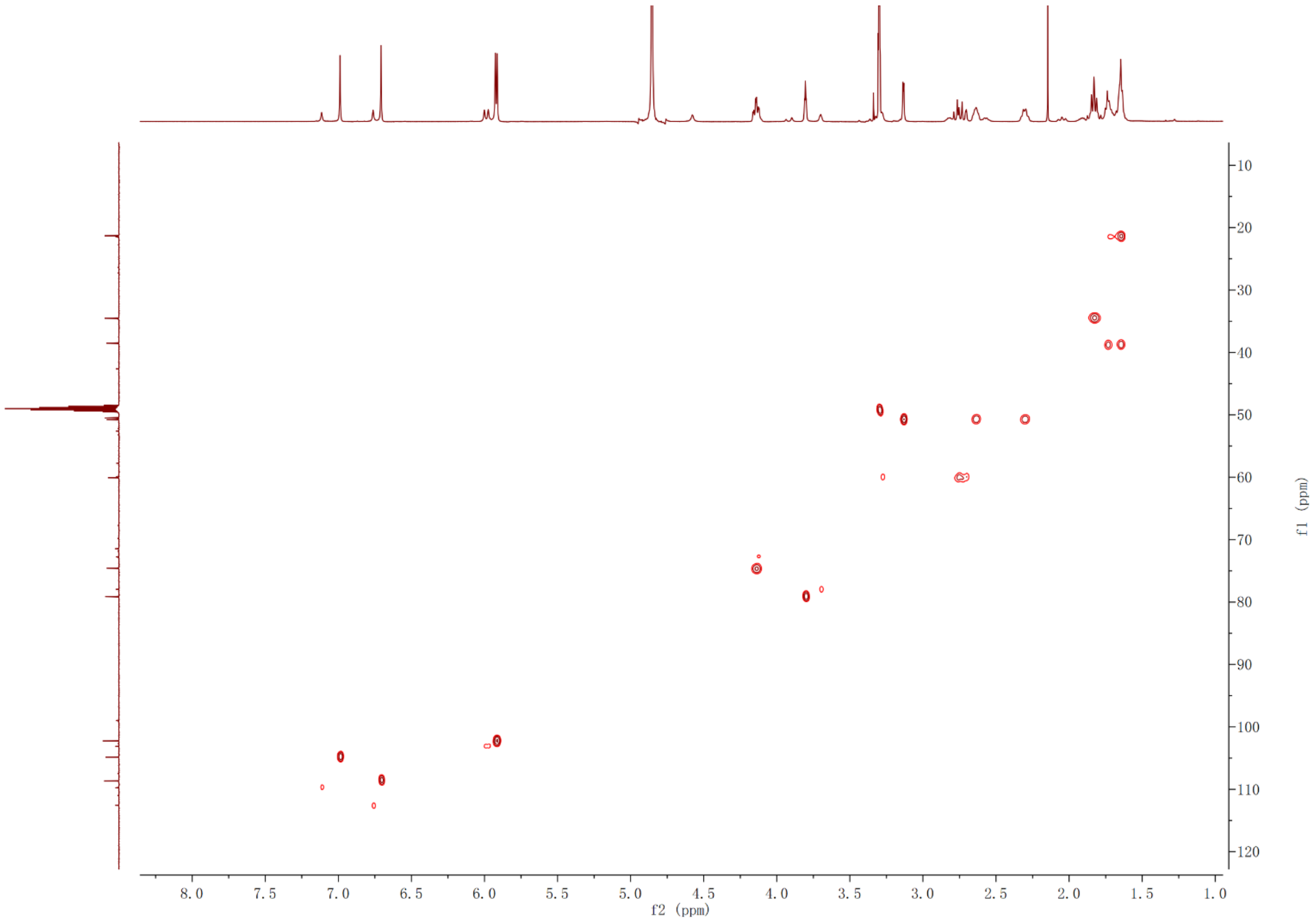

S 10 ROESY spectrum of 2 in Methanol-d_4

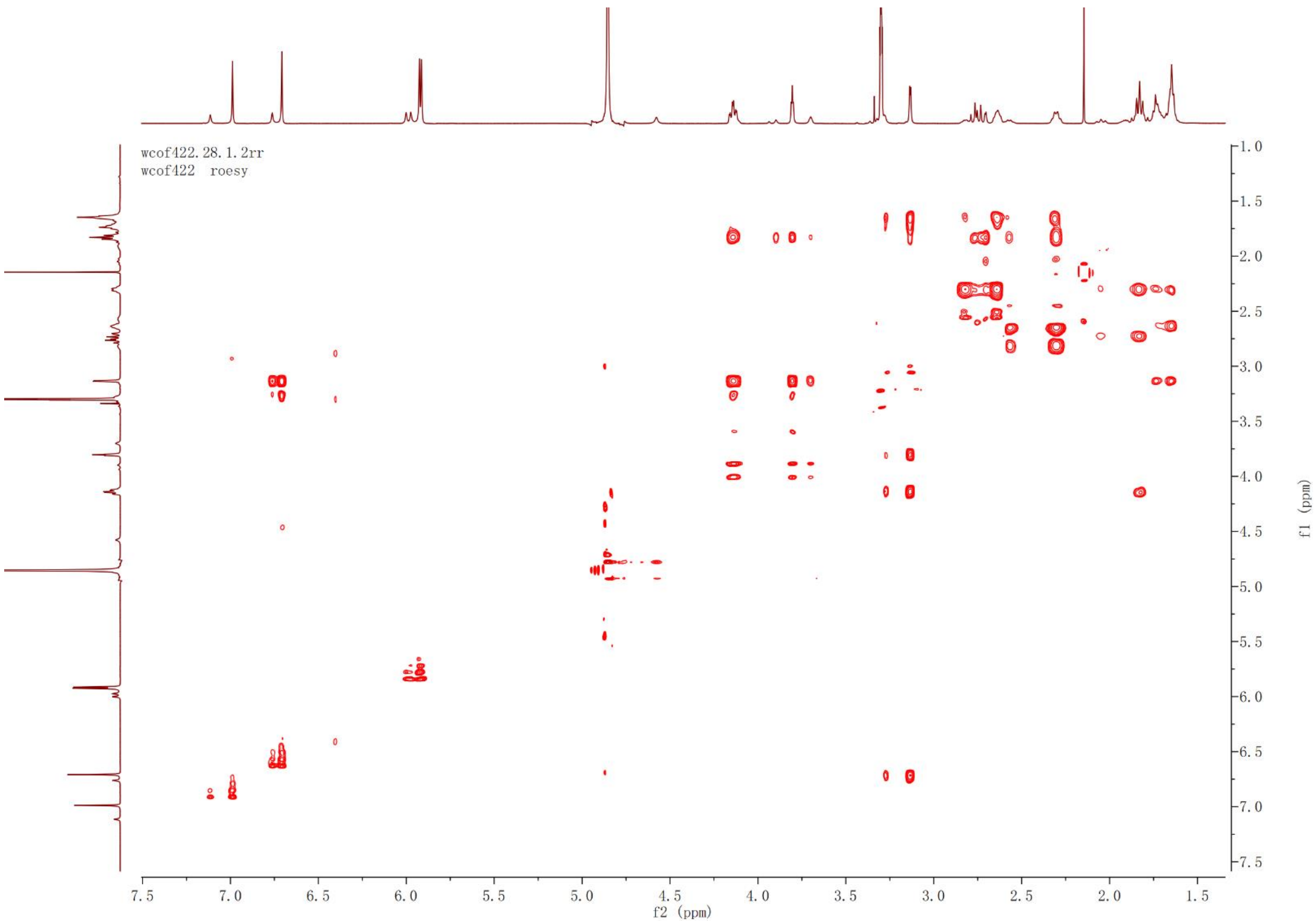

S 11 HRMS(ESI) of 2

Qualitative Analysis Report

**Data Filename**	20211119ESIA7.d	**Sample Name**	wcof42-2
**Sample Type**	Sample	**Position**	
**Instrument Name**	Agilent G6230 TOF MS	**User Name**	KIB
**Acq Method**	ESI.m	**Acquired Time**	11/19/2021 10:27:37 AM
**IRM Calibration Status**	Success	**DA Method**	ESI.m
**Comment**	+		

Sample Group **Info.**
Acquisition SW Version 6200 series TOF/6500 series Q-TOF B.05.01 (B5125.2)

User Spectra

Fragmentor Voltage	Collision Energy	Ionization Mode
200	0	ESI

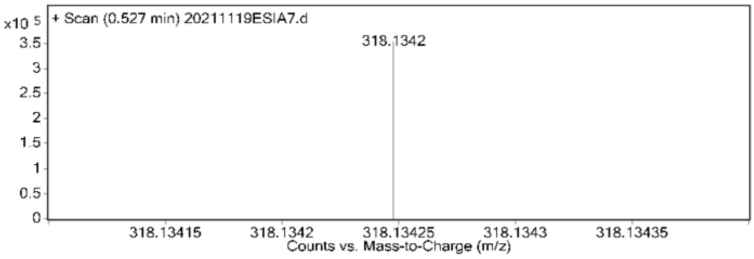

Peak List

m/z	z	Abund	Formula	Ion
318.1342	1	352948.91	C17 H20 N O5	M+
319.1389	1	129947.24	C17 H20 N O5	M+
340.1161		187069.47		
341.1214	1	110495.11		
657.2425		227334.38		
658.2479	1	278201.41		
659.2528	1	152649.77		
974.3671		60069.6		
975.3727	1	119946.59		
976.3778	1	103644.77		

Formula Calculator Element Limits

Element	Min	Max
C	0	200
H	0	400
O	0	10
N	1	1

Formula Calculator Results

Formula	CalculatedMass	Mz	Diff.(mDa)	Diff. (ppm)	DBE
C17 H20 N O5	318.1342	318.1342	-0.1	0.2	8.5

--- End Of Report ---

S 12 UV of 2 in MeOH

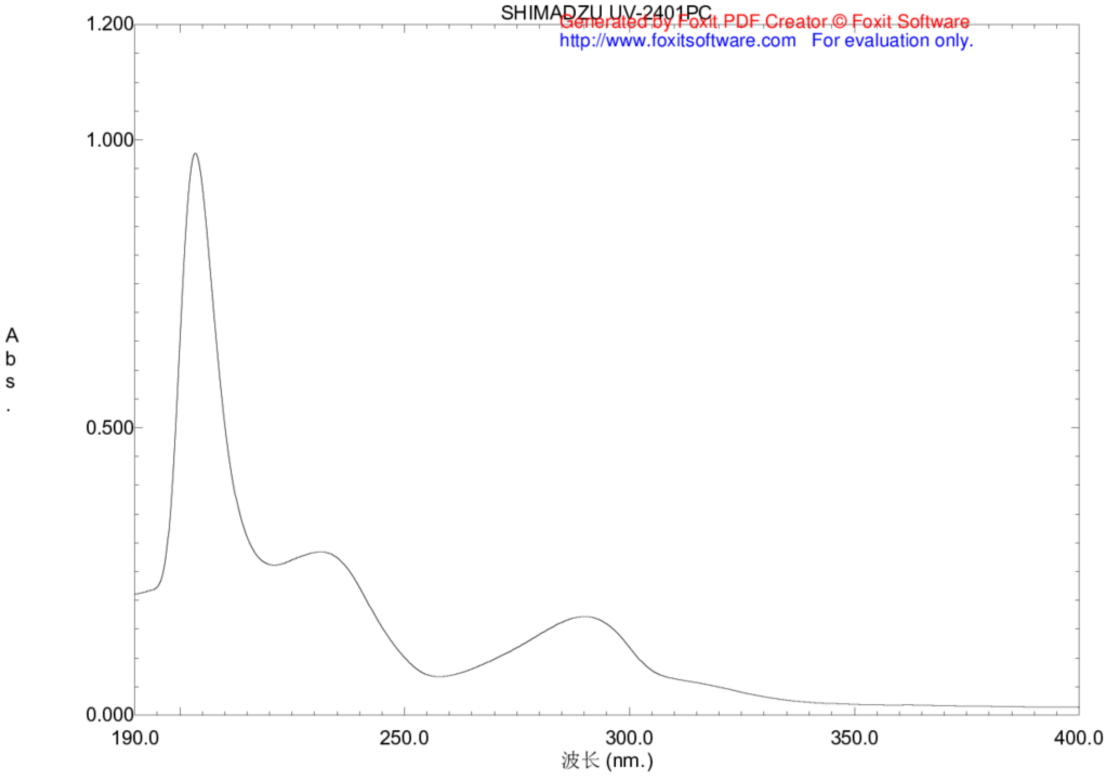

文件名: WCOF42-2

WCOF42-2

样品浓度：0.0091毫克/毫升
溶剂：甲醇

创建于: 16:16 22-03-16
数据: 原始

测量模式: Abs.
扫描速度: 中速
狭缝: 5.0
采样间隔: 0.2

否.	波长 (nm.)	Abs.
1	290.00	0.1712
2	231.20	0.2836
3	203.60	0.9762

S 13 ^{1}H NMR spectrum of 3 in Methanol-d_4

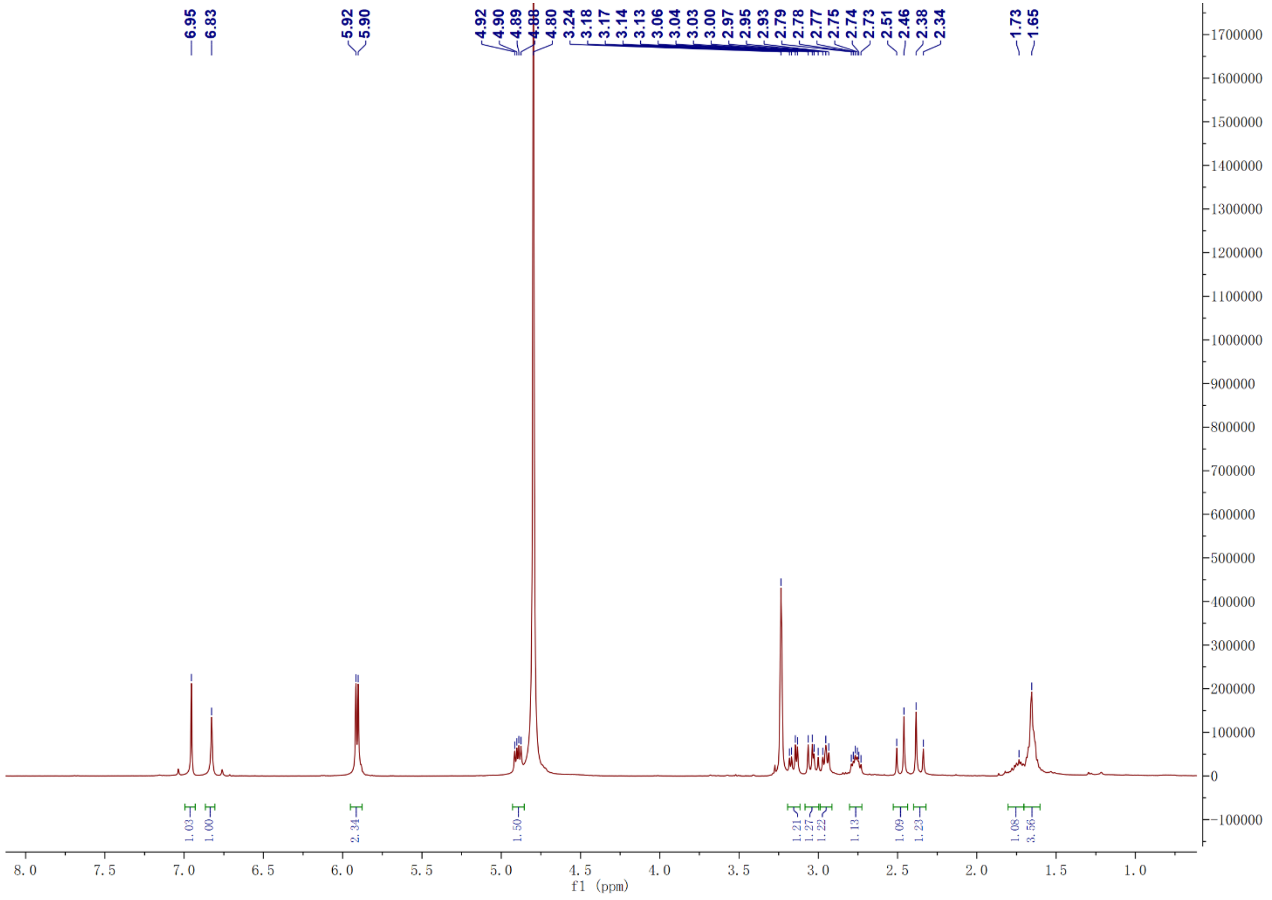

S 14 ^{13}C and DEPT spectrum of 3 in Methanol-d_4

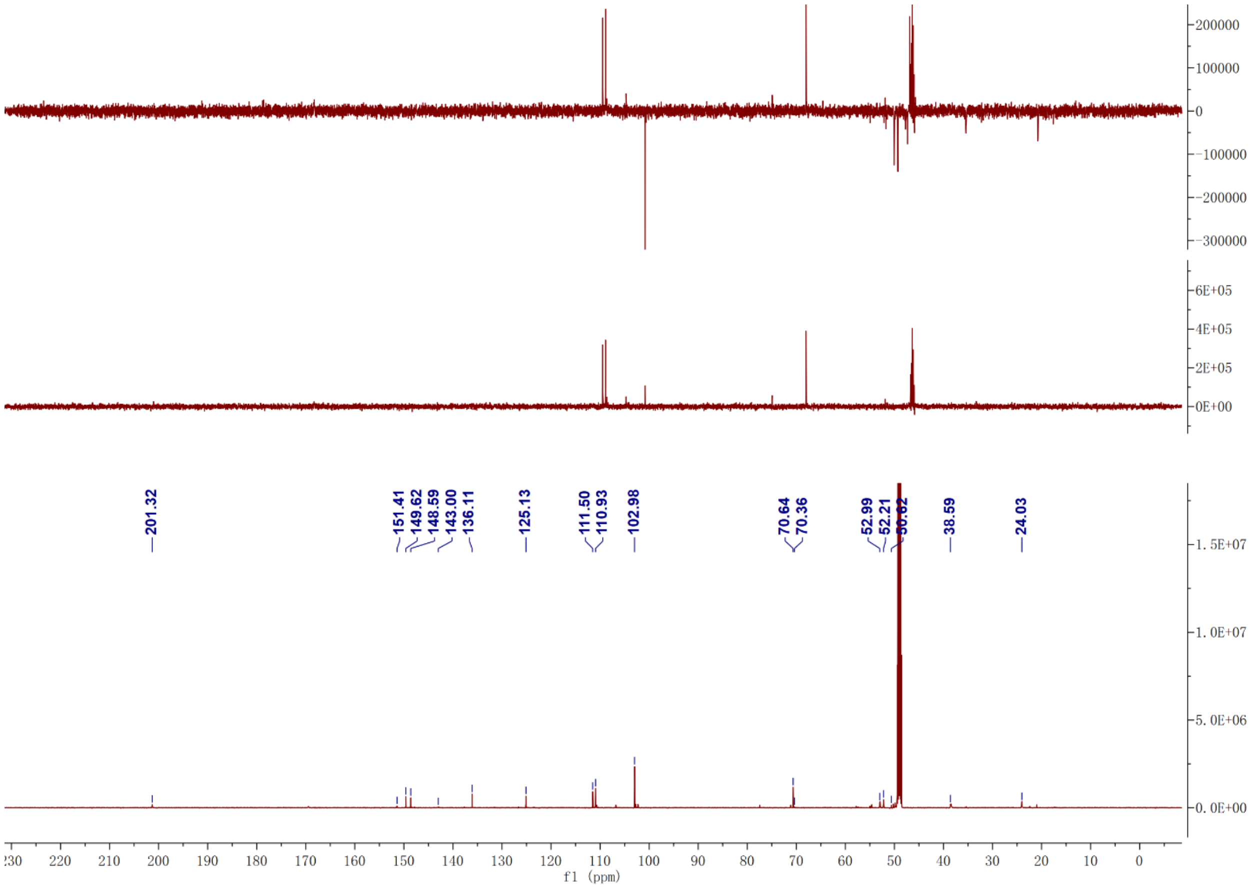

S 15 HSQC spectrum of 3 in Methanol-d_4

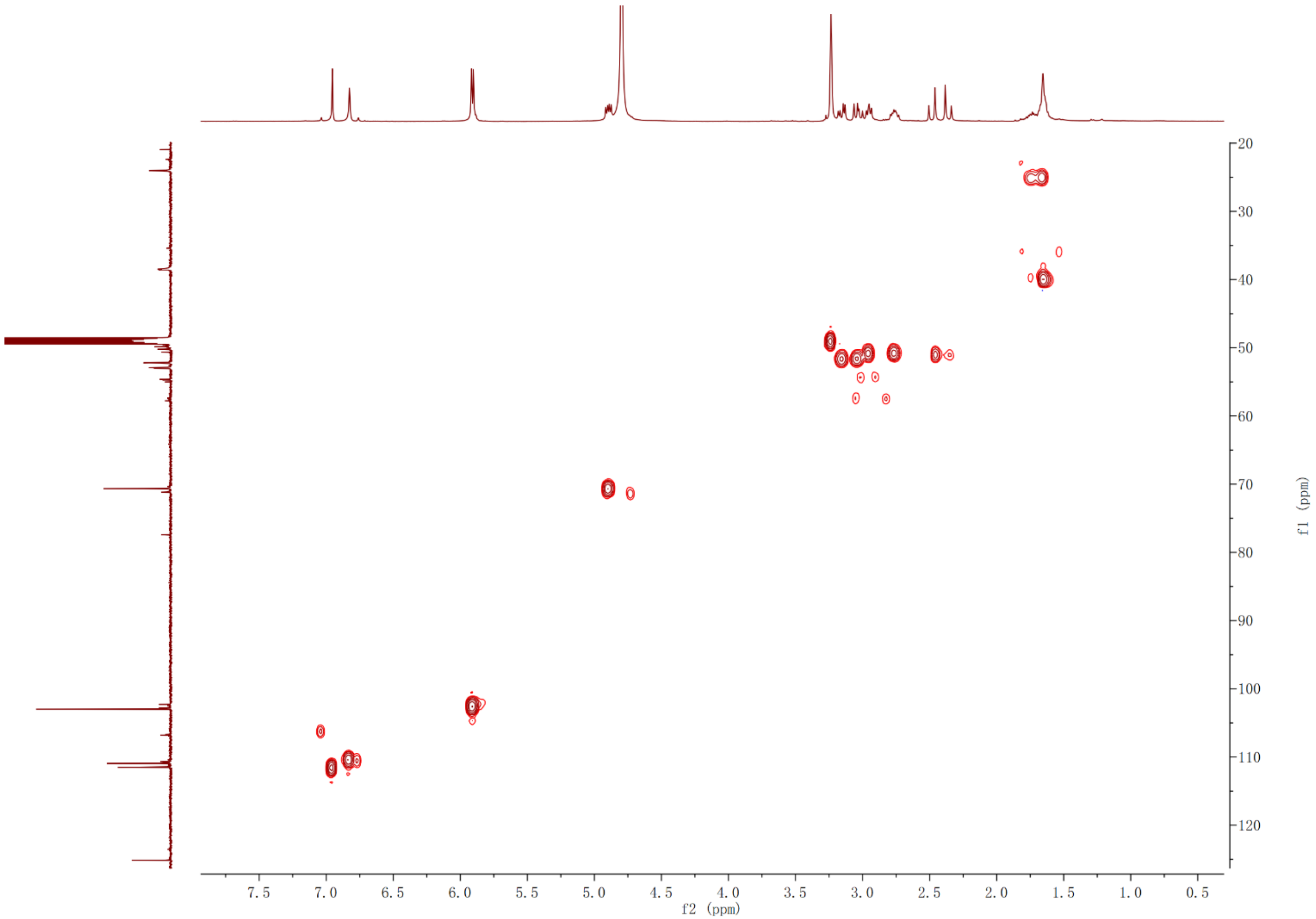

S 16 HMBC spectrum of 3 in Methanol-d_4

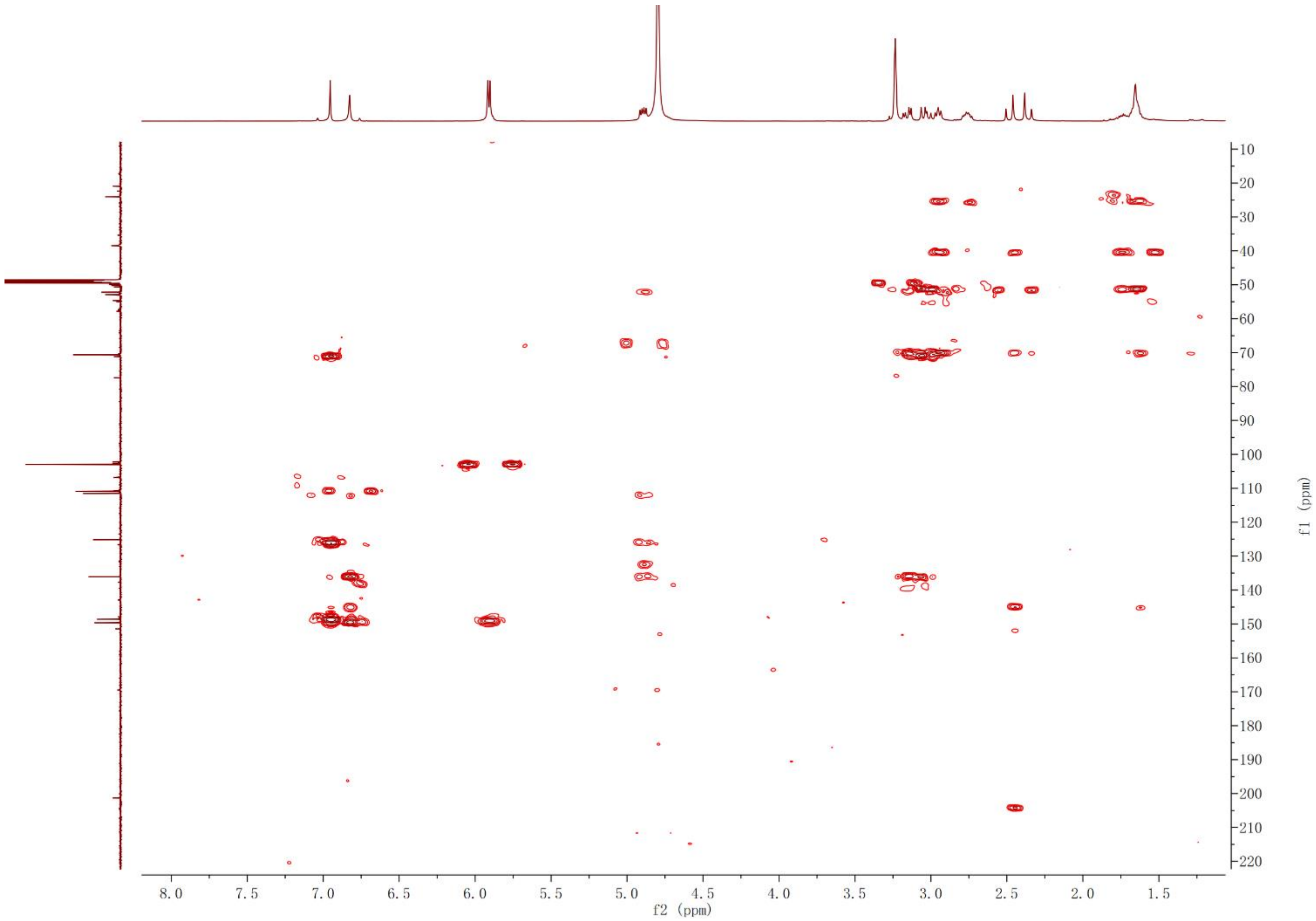

S 17 ROESY spectrum of 3 in Methanol-d_4

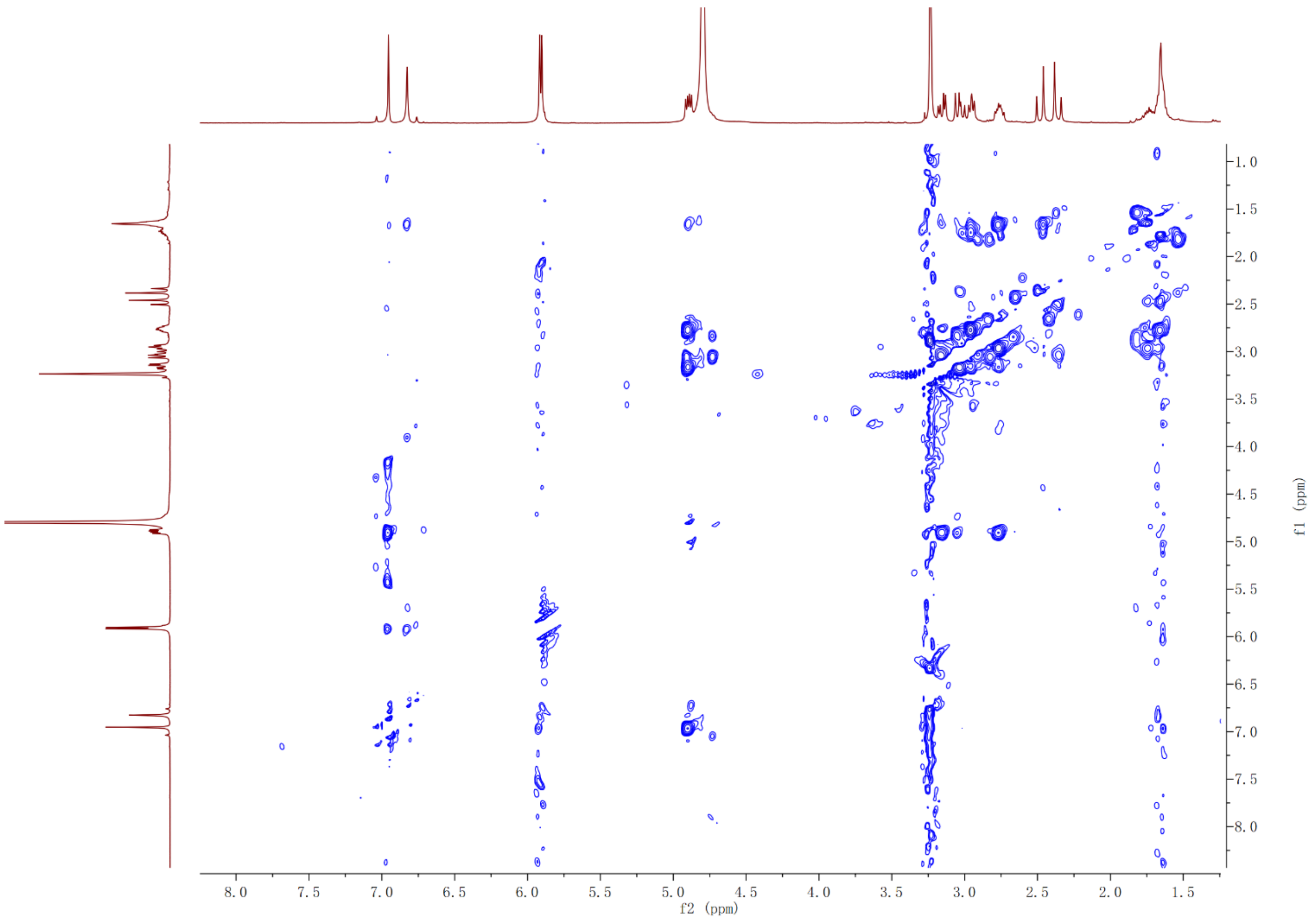

S 18 HRMS(ESI) of 3

Data File: E:\DATA\2022\0315\wcof48-1.lcd

Elmt	Val.	Min	Max	Elmt	Val.	Min	Max	Elmt	Val.	Min	Max	Elmt	Val.	Min	Max	Use Adduct
H	1	5	100	F	1	0	0	Cl	1	0	0	Ag	1	0	0	H
2H	1	0	0	Na	1	0	0	Co	2	0	0	I	3	0	0	Na
B	3	0	0	Mg	2	0	0	Cu	2	0	0	Ir	3	0	0	
C	4	5	50	Si	4	0	0	Se	2	0	0					
N	3	0	10	P	3	0	0	Br	1	0	0					
O	2	0	30	S	2	0	0	Pd	2	0	0					

Error Margin (ppm):	5	DBE Range:	not fixed	Electron Ions:	both
HC Ratio:	unlimited	Apply N Rule:	yes	Use MSn Info:	yes
Max Isotopes:	all	Isotope RI (%):	1.00	Isotope Res:	10000
MSn Iso RI (%):	75.00	MSn Logic Mode:	OR	Max Results:	20

Event#: 1 MS(E+) Ret. Time : 0.400 -> 0.440 Scan# : 61 -> 67

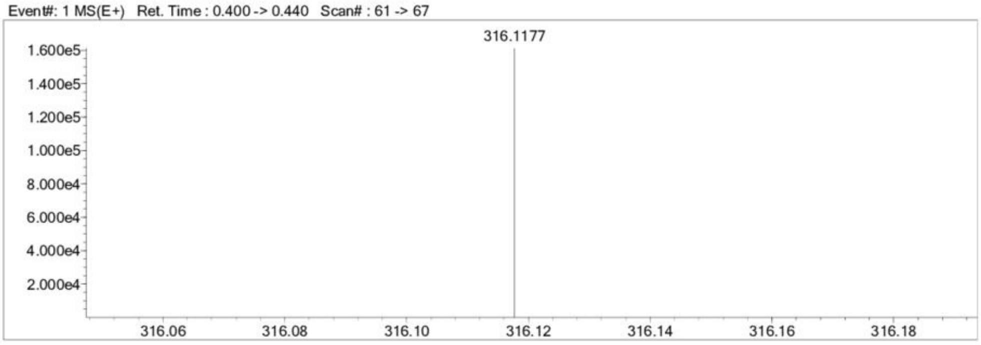

Measured region for 316.1177 m/z

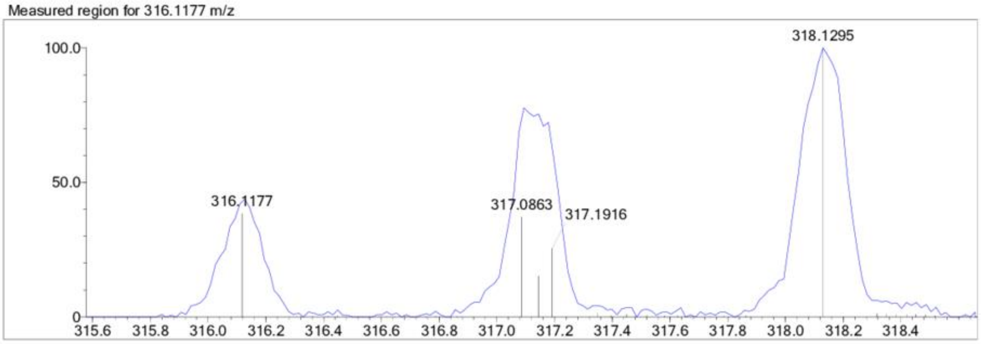

C17 H17 N O5 [M+H]+ : Predicted region for 316.1179 m/z

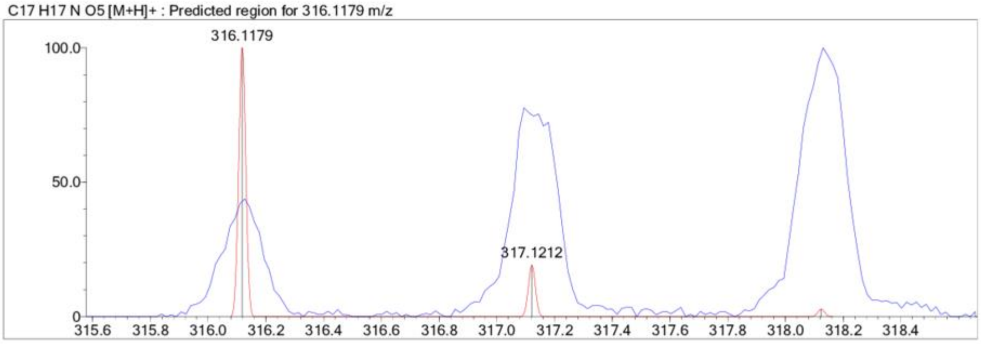

Formula (M)	Ion	Meas. m/z	Pred. m/z	Df. (mDa)	Df. (ppm)	DBE
C17 H17 N O5	[M+H]+	316.1177	316.1179	-0.2	-0.63	10.0

S 19 UV of 3 in MeOH

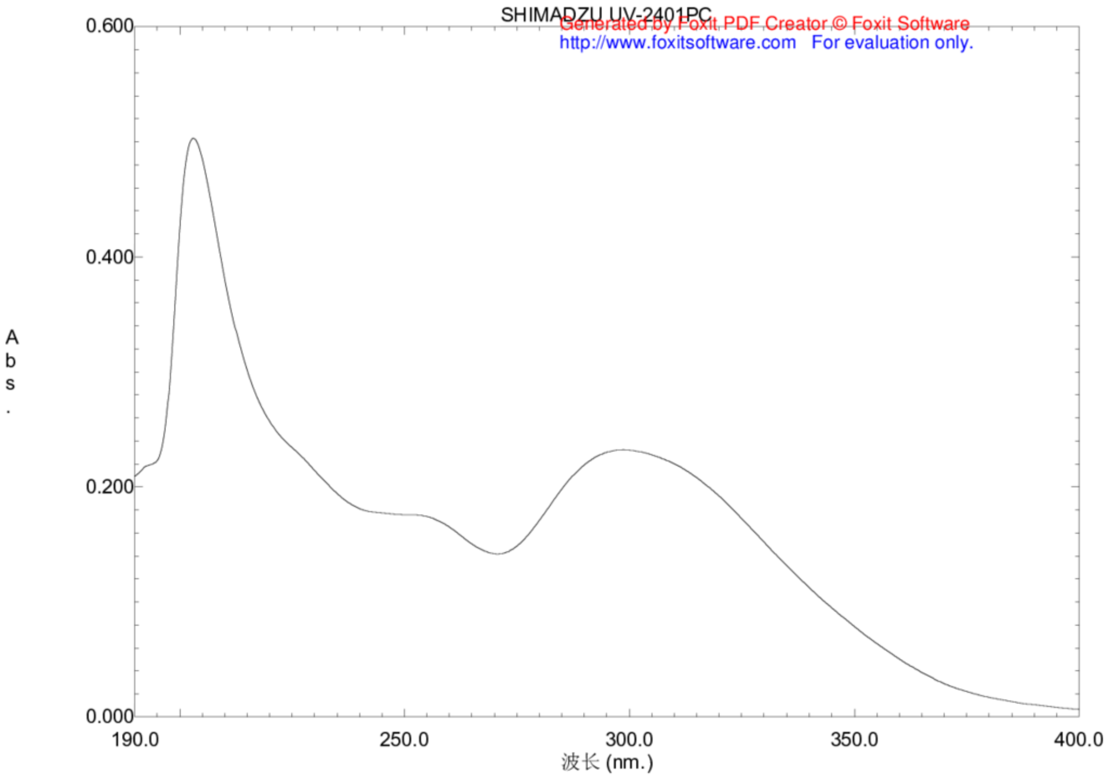

文件名: WCOF48-1

WCOF48-1

样品浓度：0.0104毫克/毫升
溶剂：甲醇

创建于: 15:47 22-03-16
数据: 原始

测量模式: Abs.
扫描速度: 中速
狭缝: 5.0
采样间隔: 0.2

否.	波长 (nm.)	Abs.
1	202.80	0.5024
2	251.40	0.1756
3	298.80	0.2320

S 20 ^{1}H NMR spectrum of 4 in Methanol-d$_4$

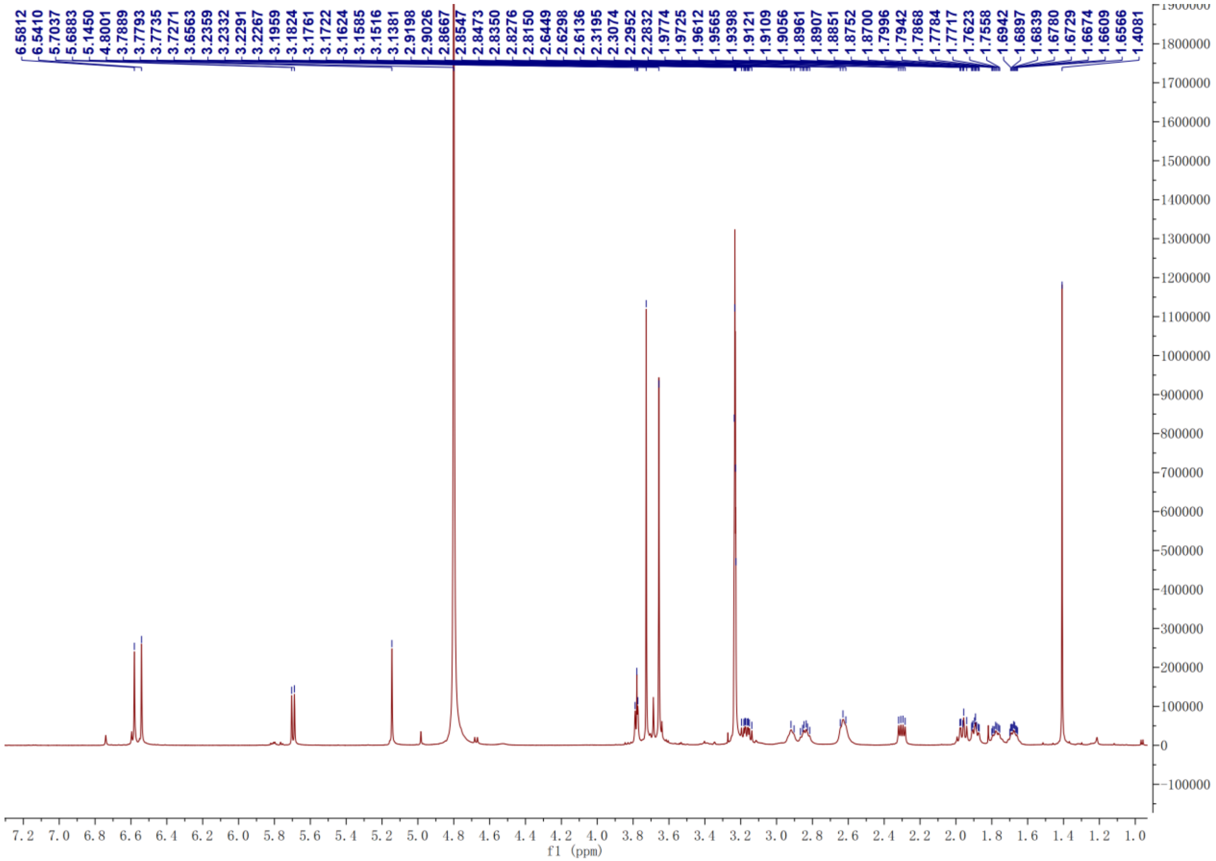

S 21 ^{13}C spectrum of 4 in Methanol-d_4

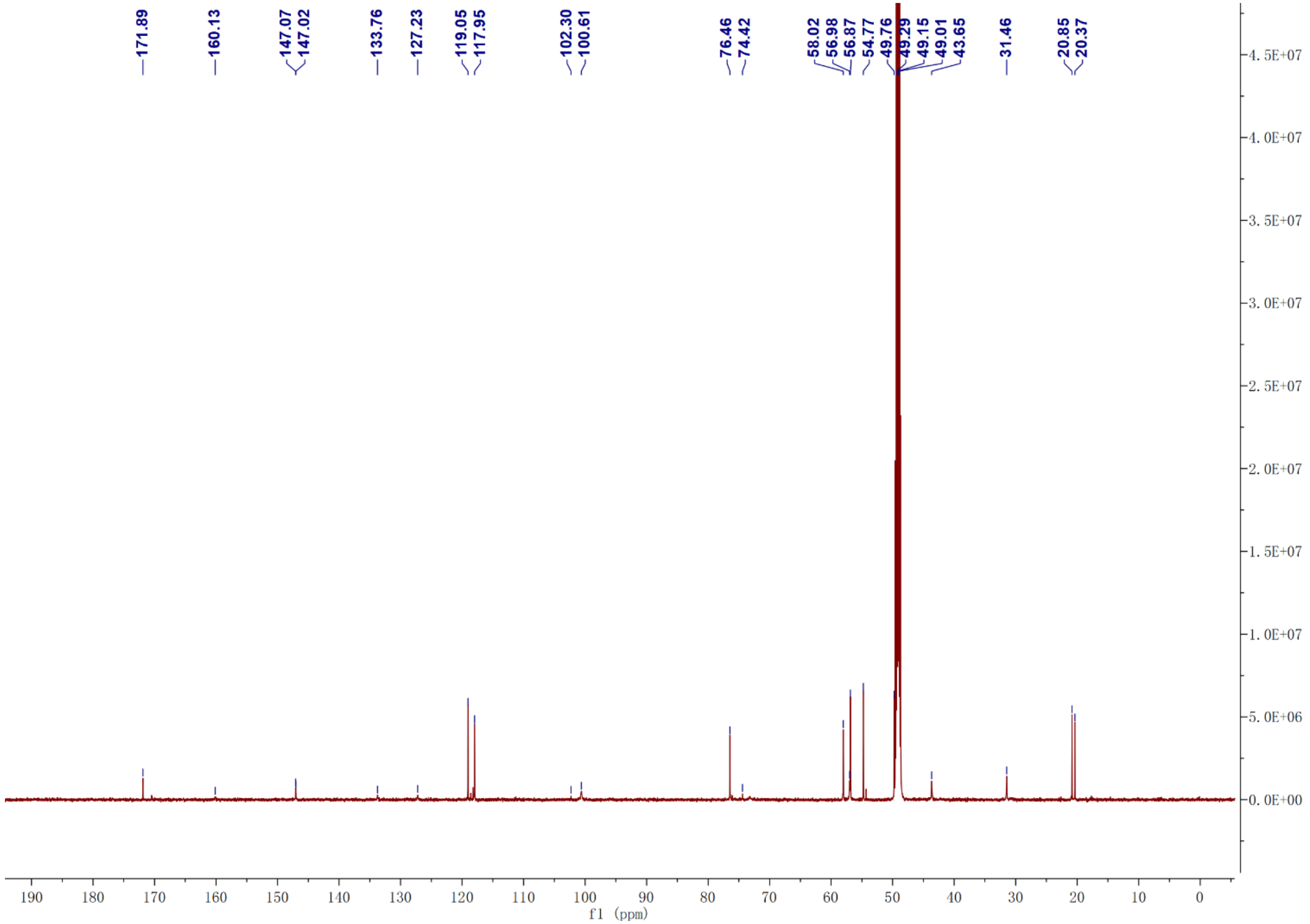

S 22 HMBC of 4 in Methanol-d_4

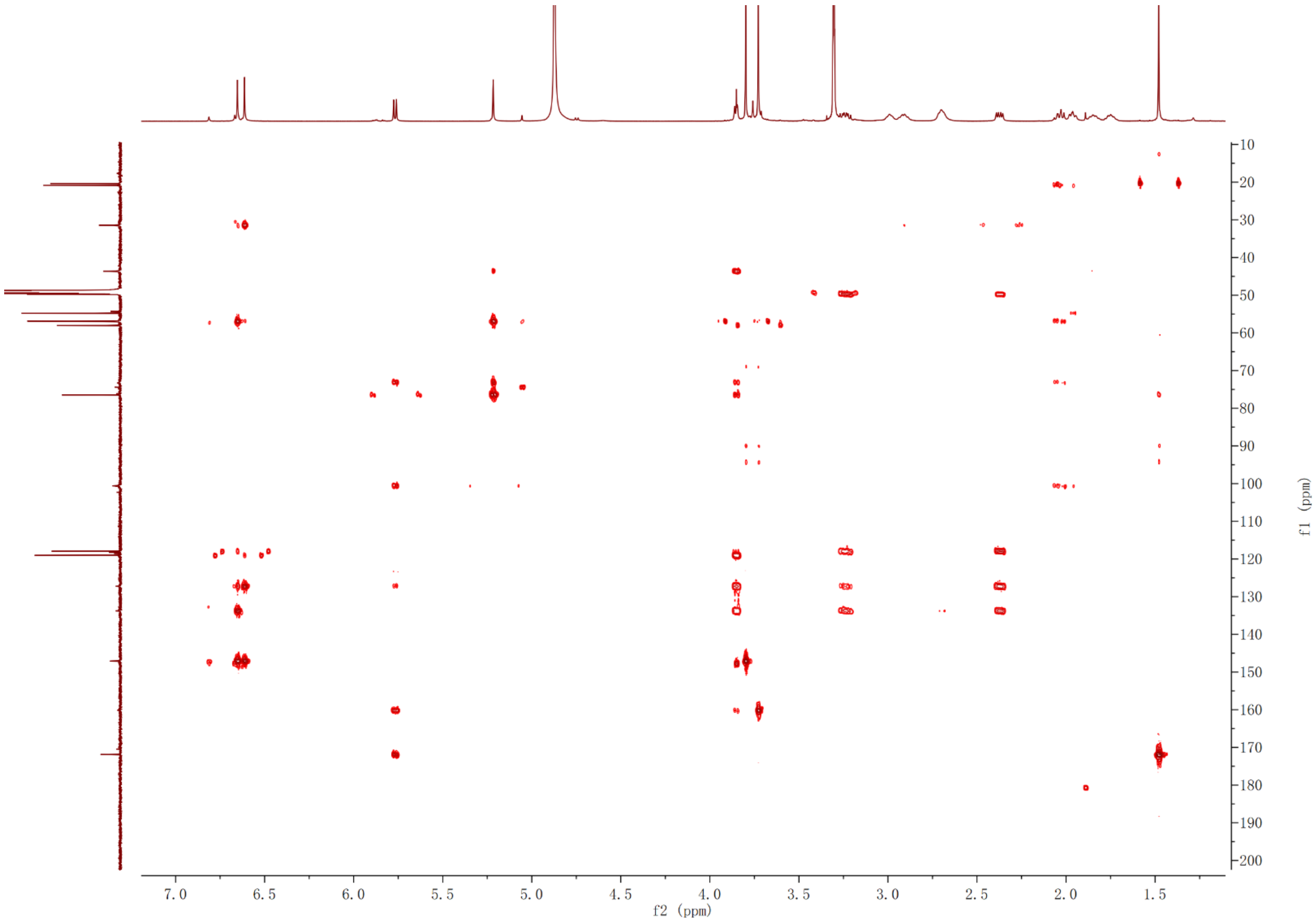

S 23 HRMS(ESI) of 4

Qualitative Analysis Report

**Data Filename**	20211119ESIA8.d	**Sample Name**	wcof50
**Sample Type**	Sample	**Position**	
**Instrument Name**	Agilent G6230 TOF MS	**User Name**	KIB
**Acq Method**	ESI.m	**Acquired Time**	11/19/2021 10:28:37 AM
**IRM Calibration Status**	Success	**DA Method**	ESI.m
**Comment**	+		

Sample Group **Info.**

Acquisition SW Version 6200 series TOF/6500 series Q-TOF B.05.01 (B5125.2)

User Spectra

Fragmentor Voltage	Collision Energy	Ionization Mode
200	0	ESI

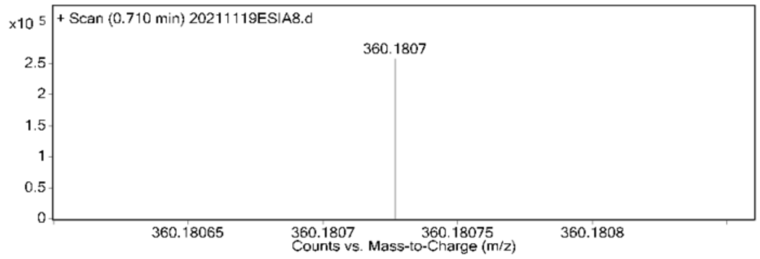

Peak List

m/z	z	Abund	Formula	Ion
121.0509	1	336544.38		
122.0532	1	24519.37		
293.1742	1	26967.22		
300.159	1	18684.61		
318.1346		30464.44		
319.1389	1	17282.21		
360.1807	1	257917.02	C20 H26 N O5	M+
361.1834	1	51920.88	C20 H26 N O5	M+
922.0098	1	478486.97		
923.0121	1	88123.91		

Formula Calculator Element Limits

Element	Min	Max
C	0	200
H	0	400
O	0	10
N	1	1

Formula Calculator Results

Formula	CalculatedMass	Mz	Diff.(mDa)	Diff. (ppm)	DBE
C20 H26 N O5	360.1811	360.1807	0.4	1.1	8.5

--- End Of Report ---

S 24 UV of 4 in MeOH

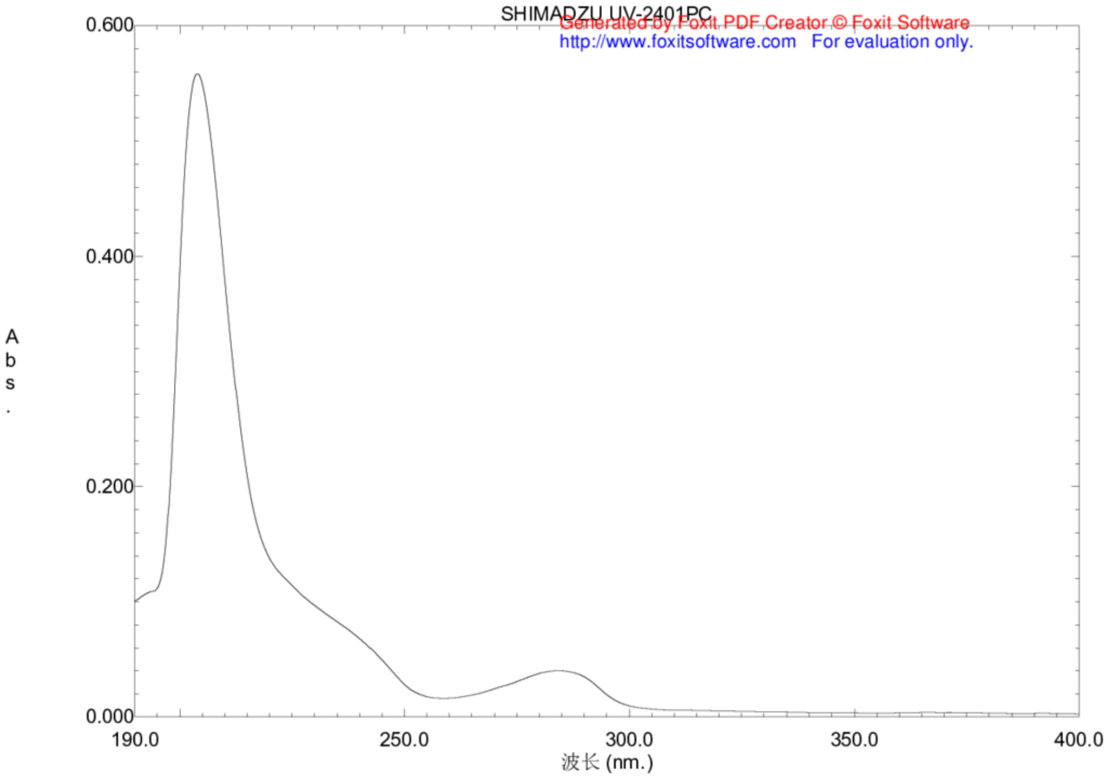

Generated by Foxit PDF Creator © Foxit Software
http://www.foxitsoftware.com For evaluation only.

文件名: WCOF50

WCOF50

样品浓度：0.0046毫克/毫升
溶剂：甲醇

创建于: 16:02 22-03-16
数据: 原始

测量模式: Abs.
扫描速度: 中速
狭缝: 5.0
采样间隔: 0.2

否.	波长 (nm.)	Abs.
1	204.20	0.5577
2	226.40	0.1087
3	283.80	0.0399

S 25 ^{1}H NMR spectrum of 5 in Methanol-d_4

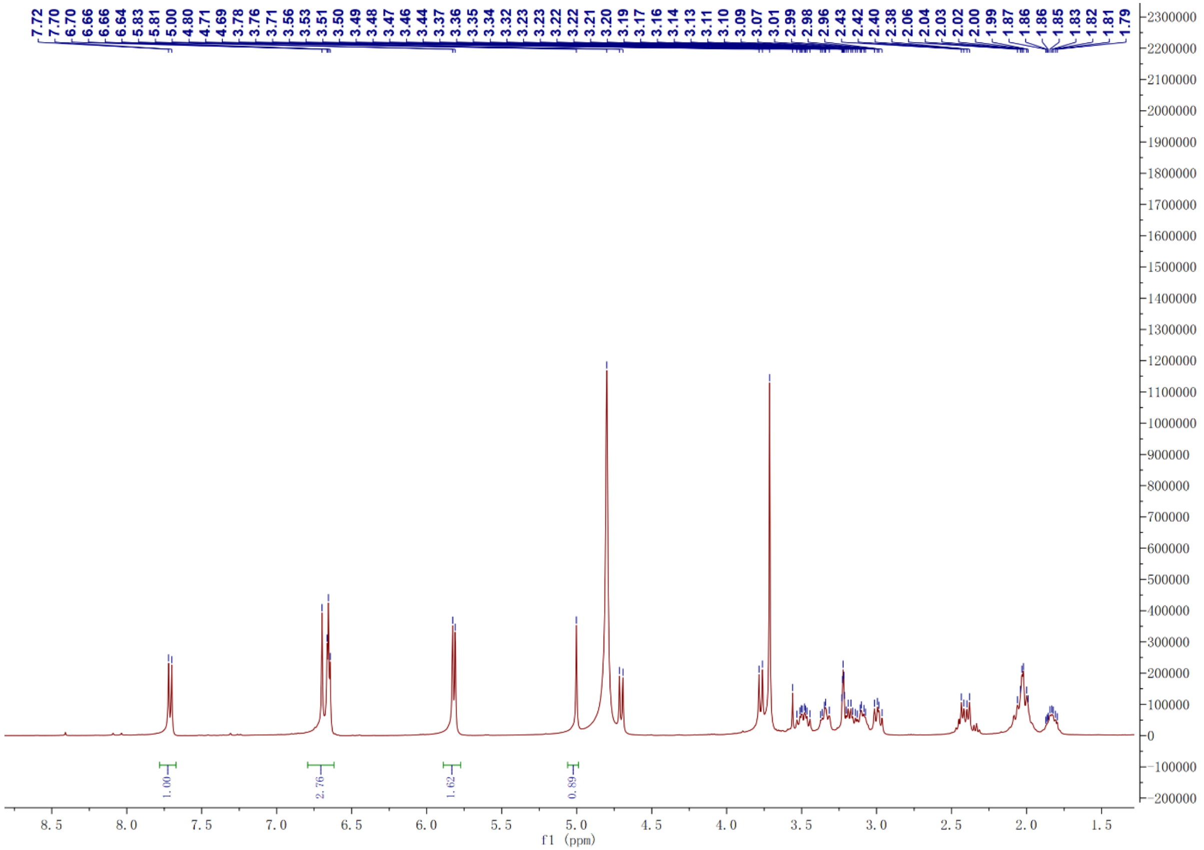

S 26 ^{13}C and DEPT spectrum of 5 in Methanol-d$_4$

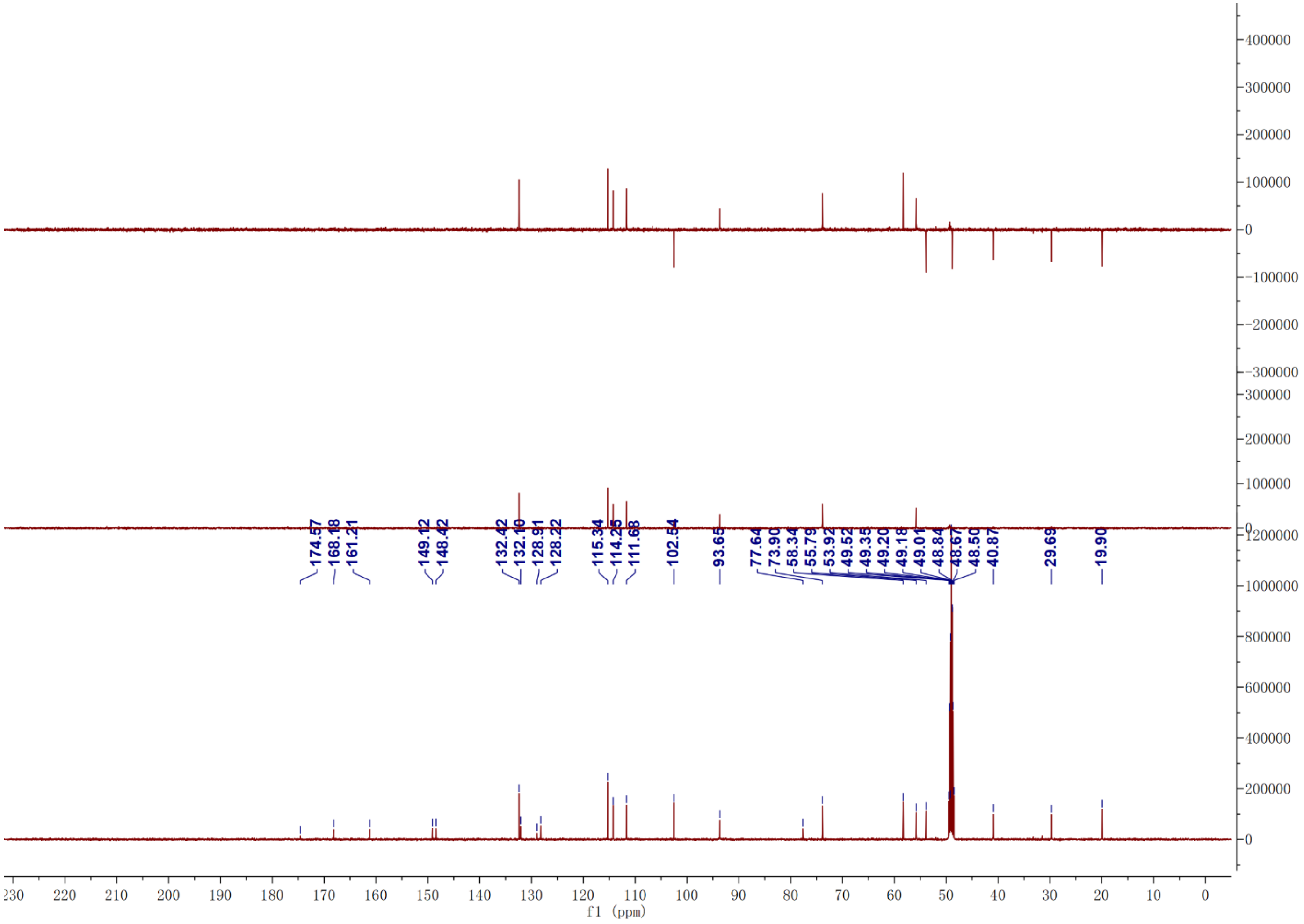

S 27 HSQC spectrum of 5 in Methanol-d_4

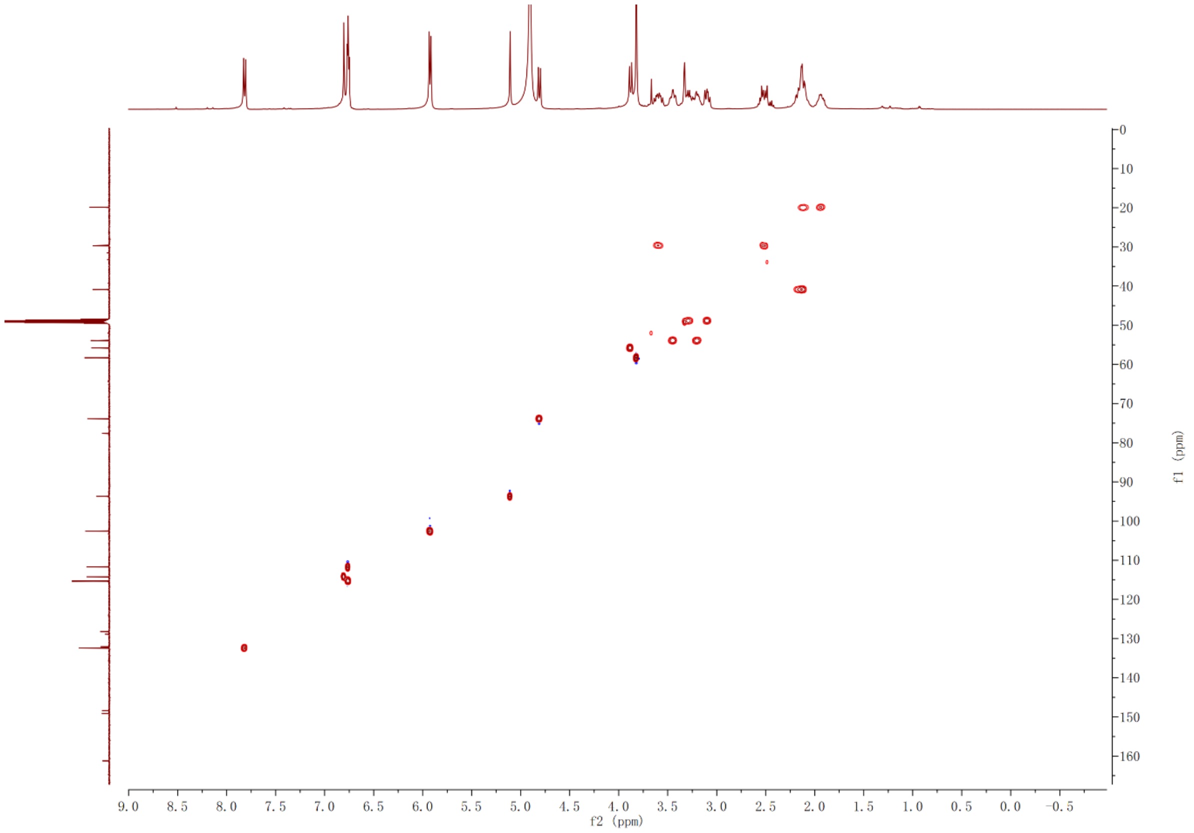

S 28 HMBC spectrum of 5 in Methanol-d_4

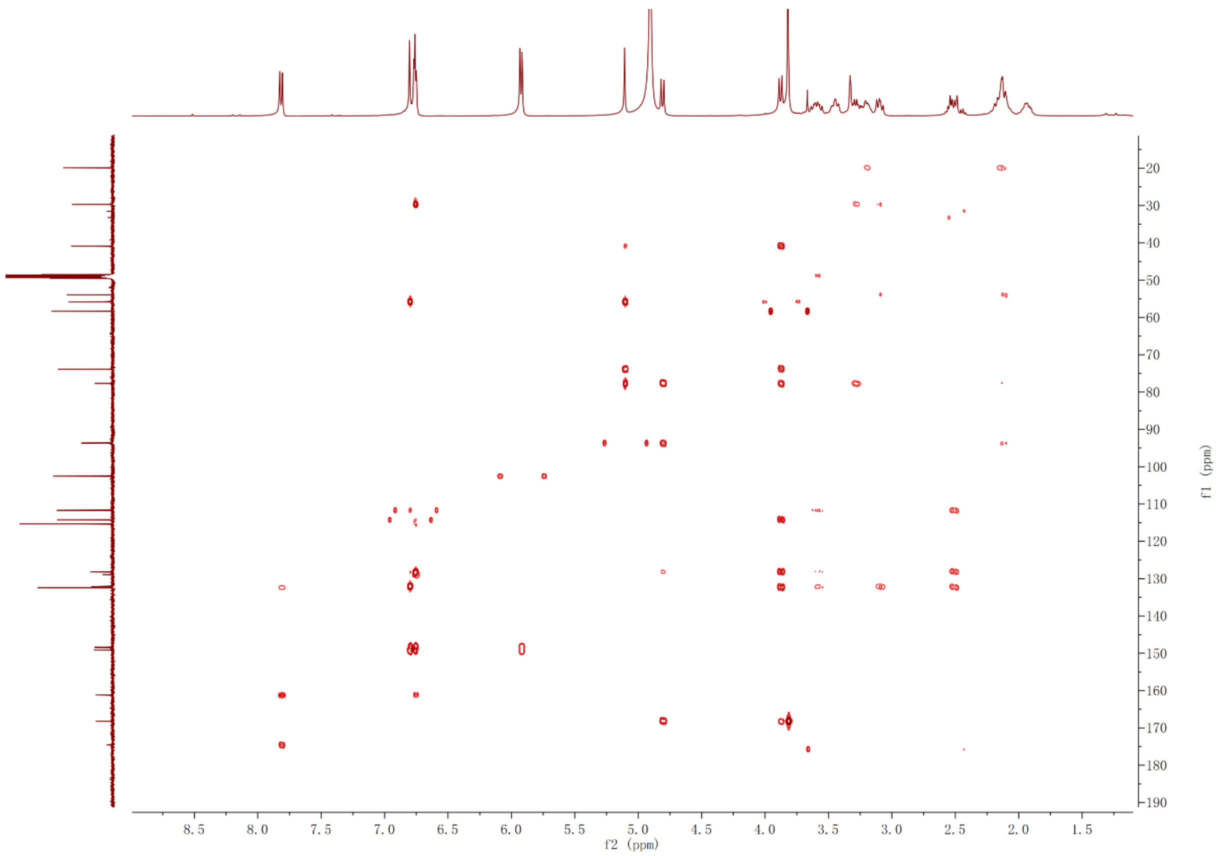

S 29 HRMS(ESI)$^{+}$ of 5

Qualitative Analysis Report

**Data Filename**	20220315ESIA1.d	**Sample Name**	wcof38
**Sample Type**	Sample	**Position**	
**Instrument Name**	Agilent G6230 TOF MS	**User Name**	KIB
**Acq Method**	ESI.m	**Acquired Time**	3/15/2022 10:29:22 AM
**IRM Calibration Status**	Success	**DA Method**	ESI.m
**Comment**			

Sample Group **Info.**
Acquisition SW Version 6200 series TOF/6500 series Q-TOF B.05.01 (B5125.2)

User Spectra

Fragmentor Voltage	Collision Energy	Ionization Mode
200	0	ESI

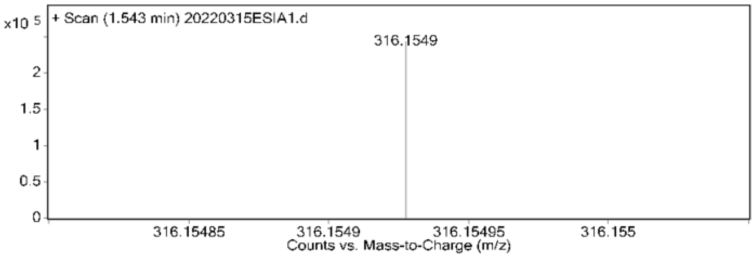

Peak List

m/z	z	Abund	Formula	Ion
102.1289	1	55125.83		
105.0434		5185.88		
242.2842	1	30167.62		
243.2879	1	5303.03		
284.1284	1	11091.13		
291.1925	1	5355.57		
316.1549	1	242777.91	C18 H22 N O4	M+
317.1576	1	53925.6	C18 H22 N O4	M+
318.1596	1	6232.26	C18 H22 N O4	M+
359.2555	1	4904.44		

Formula Calculator Element Limits

Element	Min	Max
C	0	200
H	0	400
O	0	10
N	1	1

Formula Calculator Results

Formula	CalculatedMass	Mz	Diff.(mDa)	Diff. (ppm)	DBE
C18 H22 N O4	316.1549	316.1549	0.0	0.1	8.5

--- End Of Report ---

S 30 HRMS(ESI)$^{-}$ of 5

Qualitative Analysis Report

**Data Filename**	20220315ESINA1.d	**Sample Name**	Unavailable
**Sample Type**	Unavailable	**Position**	Unavailable
**Instrument Name**	Unavailable	**User Name**	Unavailable
**Acq Method**		**Acquired Time**	Unavailable
**IRM Calibration Status**	Success	**DA Method**	ESI.m
**Comment**	Sample information is unavailable		

User Spectra

Fragmentor Voltage	Collision Energy	Ionization Mode
150	0	ESI

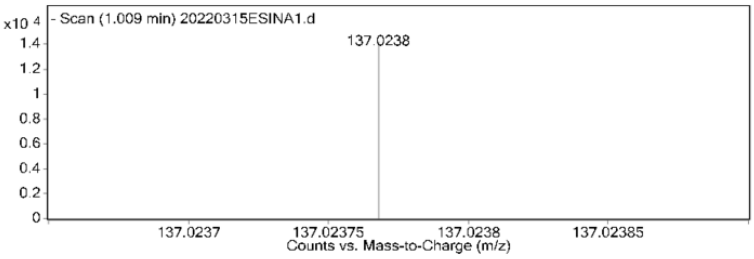

Peak List

m/z	z	Abund	Formula	Ion
112.9856		97792.82		
113.9879		265.91		
137.0238	1	14173.09	C7 H5 O3	M-
138.025	1	289.22	C7 H5 O3	M-
147.0651		227.12		
154.9726		571.92		
248.9599		464.31		
1033.9881	1	150647.98		
1034.9888	1	14101.91		
1035.9905	1	376.03		

Formula Calculator Element Limits

Element	Min	Max
C	0	200
H	0	400
O	0	10

Formula Calculator Results

Formula	CalculatedMass	Mz	Diff.(mDa)	Diff. (ppm)	DBE
C7 H5 O3	137.0239	137.0238	0.1	0.5	5.5

--- End Of Report ---

S 31 UV of 5 in MeOH

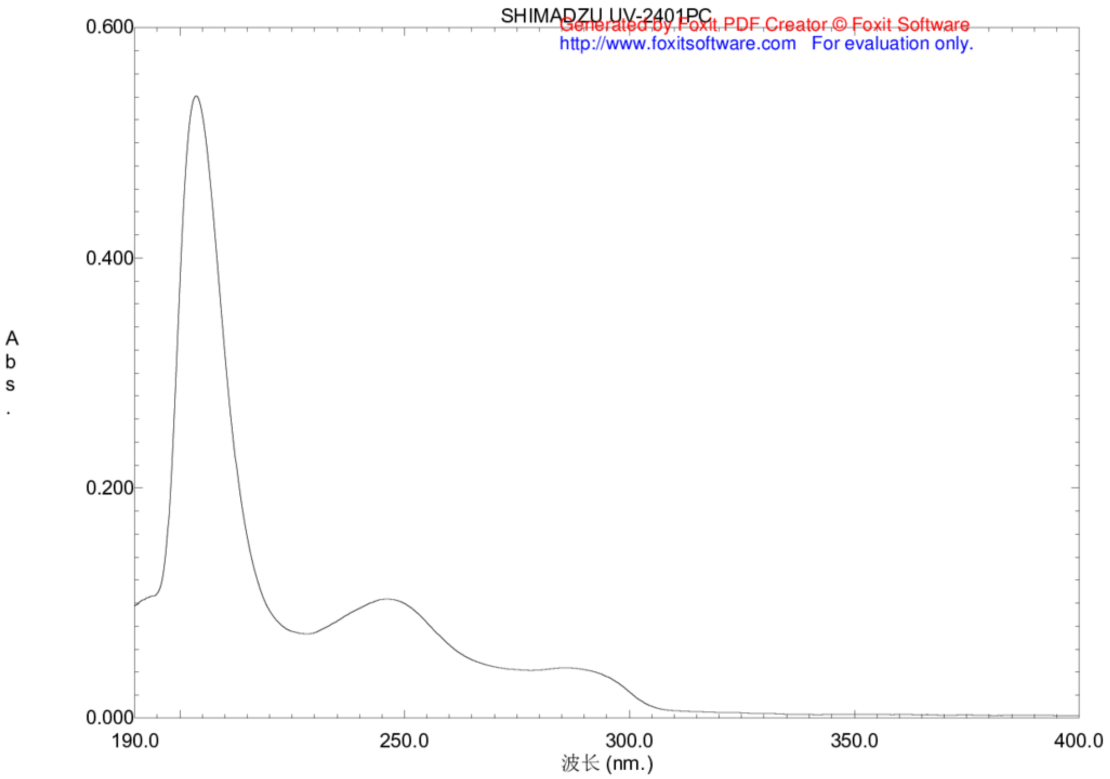

Generated by Foxit PDF Creator © Foxit Software
http://www.foxitsoftware.com For evaluation only.

文件名: WCOF38

WCOF38

样品浓度：0.0033毫克/毫升
溶剂：甲醇

创建于: 16:21 22-03-16
数据: 原始

测量模式: Abs.
扫描速度: 中速
狭缝: 5.0
采样间隔: 0.2

否.	波长 (nm.)	Abs.
1	286.20	0.0435
2	246.20	0.1032
3	203.60	0.5406

S 32 ECD Computational details of compound 4

Quantum chemical calculation

The initial conformational analysis of the compound **4** was executed by employing Monte Carlo searching algorithm via the MMFF94 molecular mechanics force field [1], with the aid of the SPARTAN'16 program package, leading to afford a panel of relatively favored conformations in an energy range of 3 kcal/mol above the global minimum. The force field minimum energy conformers thus obtained were subsequently optimized by applying the density functional theory (DFT) with the M06-2X/Def2SVP level in vacuum, implemented in the Gaussian 09 software package [2]. Harmonic vibrational frequencies were also performed to confirm no imaginary frequencies of the finally optimized conformers. These predominant conformers were subjected to theoretical calculation of ECD by utilizing Time-dependent density functional theory (TDDFT) calculations at the M06-2X/Def2SVP level in MeOH using the Polarizable Continuum Model (PCM) solvent model. The energies, oscillator strengths, and rotational strengths of each conformers were carried out with Gaussian 09 software package. The oretical calculations of ECD spectra for each conformer were then approximated by the Gaussian distribution. The final ECD spectrum of the individual conformers was summed up on the basis of Boltzmann-weighed population contribution by the SpecDisv1.71[3].

1 ECD Computational details of compound 4

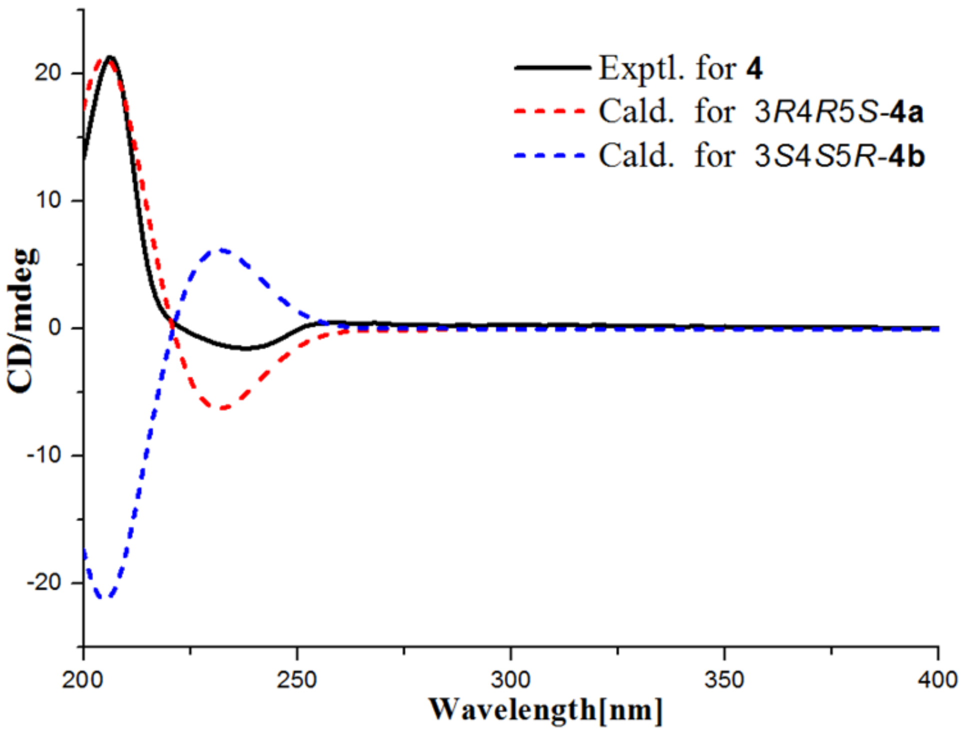

Experimental ECD spectra of **4** and calculated ECD spectra of **4a** and enantiomer-**4b** in methanol.

Table 1. Important thermodynamic parameters of the M06-2X/Def2SVP optimized conformers of **4a** in the gas phase

Conformers	E[a](Hartree)	C[b](Hartree)	G[c](kcal/mol)
4a_1	−1206. 647633	0. 379212	−1206. 268421
4a_2	−1206. 644298	0. 379157	−1206. 265141
4a_3	−1206. 642579	0. 378477	−1206. 264103
4a_4	−1206. 634679	0. 379134	−1206. 255545
4a_5	−1206. 630378	0. 378385	−1206. 251994

[a]Electronic energy; [b]Thermal correction to Gibbs free energy ; [c]Gibbs free energy (E + C).

Table 2. Conformational analysis of the M06-2X/Def2SVP optimized conformers of **4a** in the gas phase (T=298.15 K)

Conformers	ΔG (kcal/mol)[a]	Population[b]
**1a**_1	0.000000	79.48%
**1a**_2	3.377205	0.26%
**1a**_3	3.279315	0.31%
**1a**_4	0.986430	15.02%
**1a**_5	1.646560	4.93%

[a]The relative Gibbs free energy; [b]The Boltzmann distribution of each conformer.

Table 3. Cartesian coordinates for the low-energy optimized conformers of **4a** at M06-2X/Def2SVP level

Conformer 4a_1							
C	2. 322841	−0. 58694	−1. 131725	C	−4. 627867	1. 839962	−1. 084356
C	3. 324361	−0. 533926	−0. 167069	H	2. 576314	−0. 761051	−2. 178152
C	2. 988771	−0. 311395	1. 1794	H	1. 420612	0. 045401	2. 573095
C	1. 653901	−0. 147586	1. 524951	H	−0. 414123	−2. 598437	−2. 148603
C	0. 637328	−0. 202972	0. 563972	H	−1. 867036	−1. 599735	−2. 368078
C	0. 976484	−0. 417765	−0. 78109	H	−0. 730107	0. 409764	−1. 758319
C	−0. 792898	0. 021613	1. 006102	H	0. 357077	−0. 456234	−2. 837178
C	−1. 839674	−1. 051138	0. 52153	H	−2. 982005	−1. 381629	2. 372726
N	−1. 312275	−2. 079858	−0. 373199	H	−1. 41068	−2. 227	2. 266851
C	−0. 964633	−1. 738827	−1. 730305	H	−2. 92761	−4. 033892	1. 655472
C	−0. 096207	−0. 483319	−1. 835545	H	−3. 98411	−2. 926383	0. 766812
C	−2. 304398	−1. 924558	1. 699171	H	−2. 755213	−3. 448059	−1. 138454
C	−2. 935489	−3. 13755	1. 02215	H	−1. 43058	−4. 181675	−0. 214418
C	−2. 097451	−3. 303588	−0. 257711	H	−1. 548284	2. 023462	1. 527504
C	−1. 382241	1. 41393	0. 628355	H	−3. 867423	−0. 666363	−0. 47978
C	−2. 687083	1. 094745	−0. 041232	H	6. 17432	−1. 005664	−1. 68617
C	−2. 959382	−0. 217423	−0. 074714	H	4. 649348	−1. 844485	−2. 112655

O	-0.560822	2.166268	-0.254639	H	4.80571	-0.076704	-2.374691
O	4.660503	-0.680963	-0.395202	H	4.803809	-0.390403	1.667639
C	5.08299	-0.913423	-1.712238	H	2.03378	2.491461	-1.041481
O	3.958168	-0.257269	2.117528	H	0.877484	3.728082	-1.592093
C	0.461477	2.833677	0.318706	H	2.061785	4.113701	-0.278299
C	1.418539	3.345862	-0.718641	H	-5.080327	2.789332	-1.38985
O	0.583667	2.952177	1.50534	H	-4.471845	1.201845	-1.970036
H	-0.787145	-0.019232	2.102816	H	-5.302162	1.317066	-0.38584
O	-3.403	2.142233	-0.466112				

Conformer **4a**_2							
C	2.537269	0.390385	0.899783	C	-5.141673	-1.541198	-0.179128
C	3.403099	0.210933	-0.164817	H	2.938412	0.516412	1.906886
C	2.88365	0.075031	-1.466807	H	1.133368	-0.020093	-2.65207
C	1.513706	0.11091	-1.637987	H	-1.462696	1.254841	2.958978
C	0.616161	0.280724	-0.563658	H	-1.663505	-0.078462	1.826921
C	1.135561	0.429113	0.7328	H	0.567119	-0.182278	2.713659
C	-0.848703	0.177125	-0.926768	H	0.824627	1.539796	2.505585
C	-1.851122	1.249765	-0.383036	H	-2.632203	2.528249	-2.006923
N	-1.597686	1.782624	0.953007	H	-0.865764	2.542255	-1.818286
C	-1.145558	0.878702	1.970326	H	-1.409084	4.614629	-0.706497
C	0.367256	0.657673	2.025265	H	-2.886973	3.92324	0.016367
C	-1.819565	2.522775	-1.26746	H	-1.290659	3.662279	1.843079
C	-1.854721	3.700956	-0.291526	H	0.008608	3.186671	0.710389
C	-1.086567	3.132824	0.897881	H	-1.352105	-1.887378	-1.503498
C	-1.488555	-1.203681	-0.655236	H	-4.107405	0.962834	-0.212578
C	-2.935288	-0.854124	-0.446484	H	6.444122	0.198846	1.014752
C	-3.149134	0.468543	-0.37165	H	5.129062	1.24819	1.632018
O	-0.968913	-1.846058	0.507598	H	5.032194	-0.531093	1.842719
O	4.761076	0.158417	-0.094477	H	4.620037	-0.100293	-2.167934
C	5.362244	0.275845	1.16797	H	1.403968	-2.359272	1.939444
O	3.717232	-0.08625	-2.514481	H	-0.114751	-3.168566	2.417053
C	0.078909	-2.676396	0.325556	H	1.165638	-4.074942	1.514695
C	0.658676	-3.113059	1.64296	H	-5.710196	-2.476541	-0.140552
O	0.495986	-2.994264	-0.751419	H	-5.267729	-0.991231	0.768119
H	-0.908221	0.283252	-2.019564	H	-5.518161	-0.914103	-1.004478
O	-3.793852	-1.880932	-0.384374				

Conformer **4a**_3							
C	2.336692	-0.598878	-1.053663	C	-3.601131	3.10689	-0.344944
C	3.290041	-0.453913	-0.050075	H	2.6454	-0.806337	-2.07895
C	2.883657	-0.187252	1.268669	H	1.238554	0.150311	2.575711

C	1.52791	-0.075203	1.548172	H	-0.249657	-2.77723	-2.103104
C	0.55967	-0.223845	0.547759	H	-1.737048	-1.862086	-2.431663
C	0.969328	-0.479284	-0.770734	H	-0.72806	0.224087	-1.858281
C	-0.898793	-0.05315	0.917606	H	0.449357	-0.628806	-2.849207
C	-1.871796	-1.190394	0.42808	H	-3.093414	-1.496791	2.232362
N	-1.253305	-2.229069	-0.394029	H	-1.48381	-2.275257	2.242495
C	-0.860551	-1.929669	-1.748971	H	-2.899803	-4.168202	1.633512
C	-0.048683	-0.638353	-1.868793	H	-3.938206	-3.14085	0.632628
C	-2.358651	-2.035611	1.618049	H	-2.573114	-3.713974	-1.164091
C	-2.901097	-3.300751	0.961048	H	-1.271505	-4.323495	-0.123725
C	-1.979426	-3.487317	-0.256308	H	-1.739208	1.931375	1.330769
C	-1.529365	1.297077	0.457753	H	-3.869873	-0.90583	-0.688383
C	-2.790742	0.893054	-0.261123	H	6.224385	-0.863878	-1.41863
C	-2.992348	-0.428707	-0.248853	H	4.758859	-1.792508	-1.867444
O	-0.689702	2.046214	-0.419119	H	4.849116	-0.034369	-2.212603
O	4.640221	-0.546688	-0.212866	H	4.675959	-0.159947	1.839369
C	5.132434	-0.823855	-1.496927	H	1.970263	2.355745	-1.016878
O	3.805517	-0.04233	2.244119	H	0.837635	3.503033	-1.770601
C	0.287454	2.767312	0.175614	H	1.899869	4.039789	-0.405081
C	1.31181	3.217695	-0.824325	H	-4.319167	3.674396	-0.947196
O	0.320964	2.972445	1.356003	H	-3.924439	3.130748	0.708619
H	-0.941536	-0.052341	2.014321	H	-2.609916	3.575354	-0.432314
O	-3.601499	1.791041	-0.852092				

Conformer **4a**_4							
C	-2.639828	-0.686056	0.78725	C	4.802131	2.209024	-0.41541
C	-3.486617	-0.384839	-0.270734	H	-3.055042	-0.954437	1.759443
C	-2.945789	-0.073089	-1.531781	H	-1.175078	0.219439	-2.663731
C	-1.569681	-0.052385	-1.683119	H	0.207743	-2.870621	0.776184
C	-0.701594	-0.335786	-0.61607	H	0.902989	-2.66453	2.395266
C	-1.242835	-0.664431	0.632961	H	0.187457	-0.18189	2.182821
C	0.768839	-0.08826	-0.868271	H	-1.022107	-1.410559	2.613588
C	1.921686	-1.017672	-0.387788	H	2.555633	-2.012827	-2.244463
N	1.909642	-1.563394	0.98862	H	1.080595	-2.672397	-1.513715
C	0.657404	-2.125486	1.46882	H	2.398078	-4.231059	-0.365434
C	-0.373582	-1.049575	1.801803	H	3.900356	-3.436946	-0.848633
C	2.079897	-2.268774	-1.287335	H	3.946232	-2.05624	1.068478
C	2.898774	-3.255648	-0.434924	H	2.873023	-3.257637	1.793743
C	2.975365	-2.561012	0.951428	H	0.856909	2.081858	-1.239065
C	1.205156	1.345899	-0.501752	H	4.138405	-0.401033	-0.481851
C	2.69982	1.216501	-0.480482	H	-6.550387	-0.552875	0.836646
C	3.10343	-0.061002	-0.482336	H	-5.235527	-1.652039	1.358763

O	0.736147	1.746052	0.782441	H	-5.163232	0.093424	1.769729
O	-4.847131	-0.370956	-0.226431	H	-4.672408	0.157153	-2.241184
C	-5.470736	-0.635753	1.00226	H	-1.374983	1.719352	2.552148
O	-3.763383	0.201746	-2.568469	H	-0.011885	2.80562	2.915976
C	-0.402336	2.468911	0.826474	H	-1.573782	3.478393	2.296865
C	-0.865336	2.645629	2.246908	H	5.101605	1.657215	0.490888
O	-0.965146	2.890066	-0.143615	H	5.17629	1.667374	-1.300308
H	0.893492	-0.121493	-1.960978	H	5.232778	3.215771	-0.393829
O	3.405804	2.354035	-0.472064				

Conformer **4a**_5							
C	-2.57041	-0.739766	0.815331	C	3.15748	3.370852	-0.128354
C	-3.428919	-0.42586	-0.229825	H	-2.975119	-1.011565	1.790969
C	-2.901501	-0.104538	-1.494156	H	-1.142748	0.17384	-2.648692
C	-1.526701	-0.094351	-1.662665	H	0.257377	-2.949736	0.78213
C	-0.646339	-0.398747	-0.610428	H	0.974781	-2.738679	2.39038
C	-1.174975	-0.726286	0.644462	H	0.275868	-0.251052	2.172525
C	0.825869	-0.191426	-0.892563	H	-0.936912	-1.469278	2.623869
C	1.971219	-1.132244	-0.421039	H	2.558536	-2.163251	-2.273521
N	1.974109	-1.657191	0.963302	H	1.088254	-2.793751	-1.507899
C	0.722964	-2.203709	1.463288	H	2.407048	-4.349781	-0.35706
C	-0.295082	-1.117137	1.803021	H	3.909707	-3.581578	-0.880507
C	2.09599	-2.398632	-1.304811	H	4.006451	-2.16787	1.010724
C	2.918353	-3.381921	-0.451327	H	2.937415	-3.347955	1.777478
C	3.029765	-2.666184	0.921943	H	0.919754	1.960795	-1.296726
C	1.291386	1.238322	-0.557309	H	4.200242	-0.522598	-0.569336
C	2.790542	1.09046	-0.568365	H	-6.481134	-0.615163	0.905804
C	3.161717	-0.192893	-0.550057	H	-5.157732	-1.720322	1.393217
O	0.834666	1.648401	0.733	H	-5.087841	0.016682	1.83914
O	-4.788392	-0.406156	-0.169228	H	-4.635237	0.135775	-2.183765
C	-5.399822	-0.697914	1.059868	H	-1.363626	1.755007	2.356205
O	-3.729086	0.186612	-2.518231	H	0.087358	2.614812	2.921213
C	-0.178068	2.537238	0.788177	H	-1.326581	3.544469	2.279834
C	-0.725248	2.635047	2.185173	H	3.922458	4.121585	-0.355128
O	-0.579016	3.150811	-0.160782	H	2.204753	3.683256	-0.586415
H	0.928275	-0.242211	-1.986762	H	3.021432	3.305417	0.962872
O	3.62172	2.146786	-0.655489				

References

1. Halgren, T.A., 1999. MMFF VII. Characterization of MMFF94, MMFF94s, and other widely available force fields for conformational energies and for intermolecular-interaction energies and geometries. J. Comput. Chem. 20, 730-748.

https://doi.org/10.1002/(SICI)1096-987X(199905)20:73.0.CO;2-T.
2. Gaussian 09, Revision C.01, M. J. Frisch, G. W. T., H. B. Schlegel, G. E. Scuseria, ; M. A. Robb, J. R. C., G. Scalmani, V. Barone, B. Mennucci, ; G. A. Petersson, H. N., M. Caricato, X. Li, H. P. Hratchian, ; A. F. Izmaylov, J. B., G. Zheng, J. L. Sonnenberg, M. Hada, ; M. Ehara, K. T., R. Fukuda, J. Hasegawa, M. Ishida, T. Nakajima, ; Y. Honda, O. K., H. Nakai, T. Vreven, J. A. Montgomery, Jr., ; J. E. Peralta, F. O., M. Bearpark, J. J. Heyd, E. Brothers, ; K. N. Kudin, V. N. S., T. Keith, R. Kobayashi, J. Normand, ; K. Raghavachari, A. R., J. C. Burant, S. S. Iyengar, J. Tomasi, ; M. Cossi, N. R., J. M. Millam, M. Klene, J. E. Knox, J. B. Cross, ; V. Bakken, C. A., J. Jaramillo, R. Gomperts, R. E. Stratmann, ; O. Yazyev, A. J. A., R. Cammi, C. Pomelli, J. W. Ochterski, ; R. L. Martin, K. M., V. G. Zakrzewski, G. A. Voth, ; P. Salvador, J. J. D., S. Dapprich, A. D. Daniels, ; O. Farkas, J. B. F., J. V. Ortiz, J. Cioslowski, ; and D. J. Fox, G., Inc., Wallingford CT, 2010.
3. Bruhn, T., Schaumloffel, A., Hemberger, Y., Bringmann, G., 2013. SpecDis: Quantifying the Comparison of Calculated and Experimental Electronic Circular Dichroism Spectra. Chirality 25, 243-249. https://doi.org/10.1002/chir.22138.
4. Grimblat, N., Zanardi, M.M., Sarotti, A.M., 2015. Beyond DP4: an Improved Probability for the Stereochemical Assignment of Isomeric Compounds using Quantum Chemical Calculations of NMR Shifts. J. Org. Chem. 80, 12526-12534. https://doi.org/10.1021/acs.joc.5b02396.
